# A Novel lncRNA Regulates the Toll-Like Receptor Signaling Pathway and Related Immune Function by Stabilizing FOS mRNA as a Competitive Endogenous RNA

**DOI:** 10.3389/fimmu.2019.00838

**Published:** 2019-04-17

**Authors:** Hao Fan, Zengpeng Lv, Liping Gan, Chao Ning, Zhui Li, Minghui Yang, Beibei Zhang, Bochen Song, Guang Li, Dazhi Tang, Jinxin Gao, Shaojia Yan, Youli Wang, Jianfeng Liu, Yuming Guo

**Affiliations:** ^1^State key Laboratory of Animal Nutrition, College of Animal Science and Technology, China Agricultural University, Beijing, China; ^2^Key Laboratory of Animal Genetics, Breeding and Reproduction, National Engineering Laboratory for Animal Breeding, College of Animal Science and Technology, China Agricultural University, Ministry of Agriculture, Beijing, China

**Keywords:** competitive endogenous RNA, daidzein, immunoglobulins, lymphocytes, miR-548s, MAPK signaling, novel lncRNAs, Toll-like receptor pathway

## Abstract

Long non-coding RNAs (lncRNAs) have recently emerged as new regulatory molecules with diverse functions in regulating gene expression and significant roles in the immune response. However, the function of many unknown lncRNAs is still unclear. By studying the regulatory effect of daidzein (DA) on immunity, we identified a novel lncRNA with an immune regulatory function: lncRNA- XLOC_098131. *In vivo*, DA treatment upregulated the expression of lncRNA- XLOC_098131, FOS, and JUN in chickens and affected the expression of activator protein 1 (AP-1) to regulate MAPK signaling, Toll-like receptor signaling, and related mRNA expression. It also enhanced macrophage activity and increased the numbers of blood neutrophils and mononuclear cells, which can improve the body's ability to respond to stress and bacterial and viral infections. Furthermore, DA treatment also reduced B lymphocyte apoptosis and promoted the differentiation of B lymphocytes into plasma cells, which in turn resulted in the production of more immunoglobulins and the promotion of antigen presentation. *In vitro*, using HEK293FT cells, we demonstrated that mir-548s could bind to and decrease the expression of both FOS and lncRNA- XLOC_098131. LncRNA- XLOC_098131 served as a competitive endogenous RNA to stabilize FOS by competitively binding to miR-548s and thereby reducing its inhibitory effect of FOS expression. Therefore, we concluded that the novel lncRNA XLOC_098131 acts as a key regulatory molecule that can regulate the Toll-like receptor signaling pathway and related immune function by serving as a competitive endogenous RNA to stabilize FOS mRNA expression.

## Introduction

As a template RNA molecule, mRNA plays an important role in DNA genetic information transmission and protein synthesis (1). However, with advancements in research technologies, researchers have identified that mRNA accounts for only ~1.2% of transcripts, whereas non-coding RNA (ncRNA) in the transcriptome accounts for as much as 98%, which suggests that ncRNA might perform rich biological functions in organisms (2). Long non-coding RNAs (lncRNAs), which are non-coding RNA transcripts that are longer than 200 nucleotides and lack apparent open reading frames, play important roles in many cellular biological processes, including cell cycle progression, apoptosis, development, muscle differentiation, and immune regulation (3), and participate mainly in epigenetic, transcriptional, and posttranscriptional regulation (4). LncRNAs can regulate epigenetic processing by affecting chromatin reprogramming, DNA methylation and histone modification (5). In addition, lncRNAs mediate gene silencing by recruiting chromatin-modifying complexes toward target genes through sequence-specific binding (6). LncRNAs, such as Kcnq1ot1 and Air, which map to the Kcnq1 and Igf2r imprinted gene clusters, respectively, mediate the transcriptional silencing of multiple genes by interacting with chromatin and recruiting the chromatin-modifying machinery (7). Furthermore, lncRNAs can regulate gene expression at the transcriptional and posttranscriptional levels. The depletion of several ncRNAs leads to decreased expression of their neighboring protein-coding genes, including the master regulator of hematopoiesis, SCL (also called TAL1), Snai1 and Snai2 (8). After transcription, lncRNAs can regulate gene expression as competitive endogenous RNAs (ceRNAs) (5, 9, 10).

LncRNAs can participate in immune cell differentiation and the regulation of related processes. In CD8(+) T cells, some lncRNAs overlap with miRNAs and siRNAs, which suggests that lncRNAs act by being processed into smaller molecules that play a key role in the acquired immune response (11). The relationship between lncRNAs and dendritic cell differentiation was recently explained as follows: lnc-DC directly binds STAT3 in the cytoplasm and thus promotes STAT3 phosphorylation on tyrosine-705 by preventing STAT3 from binding to and being dephosphorylated by SHP1, and this phosphorylation activates the tyrosine kinase JAK/STAT signaling pathway and regulates dendritic cell differentiation (12). Recent studies revealed that in the immune system, in addition to directly regulating immune cells, lncRNAs can control inflammation by directly acting on inflammatory factors, and other studies found that lncRNAs can participate in the epigenetic modification of inflammation-related genes (13). Stimulation with lipopolysaccharide significantly increases the expression of lnc-IL7R in cells, and the expression of this lncRNA is associated with epigenetic regulation (14). In addition, lncRNA-Cox2 plays a key role in the regulation of IL-6 expression induced by Toll-like receptor stimulation (15). Multiple studies have shown that lncRNAs can be used as ceRNAs to indirectly regulate immunity-related mRNA expression through competitive binding to miRNA (16, 17). For example, let-7e regulates the inflammatory response of vascular endothelial cells by ceRNA crosstalk (18). The lncRNA XLOC_008466 regulates oncogenes in human lung cancer cells by targeting miR-874 (19), and lncRNAs play a positive role as competitive endogenous RNAs in gastric cancer (20). The involvement of lncRNAs in immune regulation is complicated, and many key immune regulatory lncRNAs have not yet been identified. Therefore, our study aimed to reveal some key novel lncRNAs in immune regulation.

Daidzein (4′,7-dihydroxyisoflavone) is a naturally occurring isoflavonic phytoestrogen belonging to the non-steroidal estrogen family and is mainly derived from leguminous plants (21). DA is also a major bioactive ingredient in the traditional Chinese medicine Gegen, which is frequently used for the treatment of fever, acute dysentery, diabetes, cardiac dysfunctions, and liver injury, among other conditions (22). The chemical structure of DA is similar to that of 17β-estradiol (E2), and DA can selectively bind different estrogen receptors (ER) at different affinities to regulate the recruitment of co-repressors and co-activators and affect ER signaling (23). Therefore, DA exerts protective effects against some diseases that are linked to the regulation of estrogen, such as breast cancer, osteoporosis, diabetes, and cardiovascular diseases (24). DA also has several other biological activities that are independent of the ER, such as anti-inflammation and anticancer activities and protection of the skin and nerves. These beneficial effects are mainly due to the regulation of the immune response (25). DA has been shown to precisely orchestrate processes related to the regulation of the secretion of immune molecules, the proliferation and differentiation of immune cells, and immune signal pathways. Feeding mice DA after the onset of experimental allergic encephalomyelitis reduced the secretion of interferon-3 and interleukin-12, enhanced interleukin-10 production, suppressed lymphocyte proliferation, and decreased cytotoxicity (26). Studies have shown that under conditions of LPS-induced inflammation, DA could inhibit inflammatory hyperreactivity and eliminate inflammation by inhibiting the differentiation of B lymphocytes (27). In addition, mice treated with DA exhibited increased percentages of CD4(+) and CD28(+) T cells, and DA treatment regulated B lymphopoiesis and decreased the mRNA levels of RANKL in B220(+) cells (28). DA can affect the immune response by regulating the Toll-like and NF-κB pathways. We thus attempted to identify key lncRNAs involved in immune regulation by studying the daidzein-mediated changes in lncRNA expression associated with the regulation of the immune response (29, 30).

Numerous studies have shown that plant extracts not only regulate the expression of coding genes but also exert regulatory effects on non-coding genes (31–34). Although these studies highlight the relevance of lncRNAs in the immune regulation process, the precise molecular mechanisms remain largely unelucidated. Furthermore, the role of lncRNAs involved in the regulation of the immune response to DA remains unknown. In this study, we treated egg-laying hens with DA, screened for key mRNAs and lncRNAs related to immune regulation using transcriptome technology, and performed cell experiments to explore the specific molecular biological mechanisms underlying the regulation of immune responses by lncRNAs.

## Materials and Methods

### Materials

The DA used in this study was synthetically produced by the Kai Meng Co. (Xi An, Shanxi, China) Chemical Plant with a purity of 99.9%.

### Feeding Experimental Design and Bird Management

The experimental animal procedures were approved by the China Agricultural University Animal Care and Use Committee (Beijing, China, permit number SYXK20130013). The experiment was performed with laying broiler breeder hens housed at a commercial farm (Zhuozhou, China) under standard conditions. After a 2-week acclimation period, a total of 480 57-week-old Ross 308 laying broiler breeder hens were allocated to two treatment groups: the DA-deficient group (DAD), which the diet is specially formulated lacking DA, and the DA-supplemented group (DS). Each treatment was replicated eight times, and each replicate included 30 broiler breeder hens. The hens were fed a nutritionally balanced corn-miscellaneous meal (CSCM) with DA added at 0 and 20 mg/kg for 8 weeks. The CSCM diets were formulated to meet the nutrient requirements of laying broiler breeders according to the NRC guidelines (1994) (Table 1).

**Table 1:** Composition and nutritional levels in the experimental diets of hens.

**Ingredient (%)**	**DAD group diet**	**DS group diet**
Corn	68.99	68.99
Soybean meal	4	4
Corn protein	9.15	9.15
De-gossypol cottonseed protein	6	6
Limestone	7.76	7.76
Soybean oil	0.5	0.5
Dicalcium phosphate	2.09	2.09
NaCl	0.35	0.35
^a^Trace mineral premix	0.3	0.3
Choline chloride (50%)	0.12	0.12
Mycotoxin adsorbent	0.1	0.1
DL-methionine	0.0515	0.0515
^b^Vitamin premix	0.035	0.035
Santoquin	0.03	0.03
Phytase	0.016	0.016
4% Flavomycin	0.015	0.015
Lysine•HCl (8%)	0.373	0.373
Threonine	0.0664	0.0664
Tryptophan	0.0481	0.0481
Daidzein	0	20 ppm
**Total**	100	100
Avian metabolic energy (MC/kg)	2.83	2.83
Crude protein (%)	16.1	16.1
Calcium (%)	3.48	3.48
Total phosphorus (%)	0.678	0.678
Available phosphorus (%)	0.47	0.47
Methionine (%)	0.34	0.34
Lysine (%)	0.805	0.805
Met+Cys (%)	0.626	0.626
Threonine (%)	0.6	0.6
Tryptophan (%)	0.18	0.18

a *Supplied the following per kg complete diet: Cu, 8 mg; Zn, 75 mg; Fe, 80 mg; Mn, 100 mg; Se, 0.15 mg; I, 0.35 mg*.

b *Supplied the following per kg complete diet: vitamin A, 12,500 IU; vitamin D3, 2,500 IU; vitamin E, 30 IU; vitamin K3, 2.65 mg; thiamine, 2 mg; riboflavin, 6 mg; vitamin B12, 0.025 mg; biotin, 0.0325 mg; folic acid, 1.25 mg; pantothenic acid, 12 mg; niacin, 50 mg*.

### Sample Collection and Chemical Analysis

At the 4 and 8th weeks of the experiment, one chicken per replicate was selected and deprived of food for 8 h. One blood sample was collected from the wing vein of each replicate chick into vacuum blood collection tubes, and the serum was centrifuged at 3000 × g for 15 min and stored at −20°C until use for the detection of immunoglobulins (Igs). Another blood sample was collected from the wing vein of each replicate chick into vacuum blood collection tubes (with heparin sodium) for assessing lymphocyte proliferation and percentages. At the 8th week of the experiment, one chicken from each replicate was slaughtered. One liver sample was immediately collected from each replicate for measuring gene expression, and the samples were frozen in liquid nitrogen and stored in a freezer at −80°C.

### Serum Immunoglobulin Levels

The serum IgM and IgA levels were determined using a commercial ELISA kit (IDEXX laboratories Inc., Westbrook, Maine, USA) according to the manufacturer's recommended protocol.

### Lymphocyte Classification and Proliferation

Peripheral blood mononuclear cells (PBMCs) were isolated by Ficoll density centrifugation. Briefly, heparinized blood was diluted with Hank's balanced salt solution at a ratio of 1:1 (no calcium and no magnesium, Life Technologies, Burlington, Vermont, USA) and carefully layered on top of Histopaque 1077 (Sigma-Aldrich Corporation, Burlington, Vermont, USA) in a 10-mL centrifuge tube at a 2:1 ratio. After centrifugation for 30 min at 3,000 rpm and 20°C, the PBMCs at the plasma-Ficoll interface were collected and washed three times with cold RPMI-1640 medium (containing 5.0% inactivated fetal bovine serum, 0.0599 mg/mL penicillin, 100 μg/mL streptomycin, and 24 mM HEPES) by centrifugation at 1,800 rpm and 4°C for 10 min. The cell counts and viability were evaluated by trypan blue staining. The lymphocytes were then mixed with CD3 (SPRD), CD4 (FITC), and CD8 (RPE) antibodies or Bu-1 (RPE) antibodies, and the cells were then incubated in a water bath at 37°C for 30 min, washed twice with Hanks solution and fixed with 3% paraformaldehyde. The results are expressed as percentages. The proliferative responses of T and B cells after stimulation with concanavalin A (ConA, 45 μg/mL) and LPS (25 μg/mL), respectively, were determined by an MTT assay. ConA from Canavalia ensiformis (C2010) and LPS from Escherichia coli (L2880) were both obtained from Sigma-Aldrich Corporation. The results are expressed as SI values (35).

### Blood Routine Examination

The routine blood examination was performed at Xiyuan Hospital, Beijing, China.

### Next-Generation Sequencing (NGS)

Total RNA samples for sequencing were purified from 20 mg of tissue samples from eight chickens (four replicates in each treatment group) using the RNeasy Fibrous Tissue Mini messenger RNA (mRNA) extraction kit (Qiagen, Hilden, Germany) following the manufacturer's recommendations. The concentration and purity of total RNA were determined using a UV/Vis spectrophotometer (ACTGene, New Jersey, USA) at 260 nm, and the sample integrity was evaluated through a microfluidic assay using a Bioanalyzer system (Agilent Technologies, Inc., Santa Clara, California, USA). Only high-quality RNA extracts [RNA integrity number (RIN) ≥ 8] were used to pool equal amounts of RNA per chicken within each treatment group, and NGS data were obtained from the pooled RNA samples from each group to ensure the most robust transcriptome. Complementary DNA (cDNA) libraries for RNA sequencing (RNA-Seq) were constructed using a TruSeq RNA Sample Prep Kit v2 (Illumina, San Diego, California, USA), and RNA-Seq analysis was performed to identify transcriptional changes using a MiSeq instrument (Illumina) with paired-end libraries (CapitalBio, http://cn.capitalbio.com/) (36). Four replicates from each treatment were analyzed independently for library synthesis and sequencing, and the quality of the raw reads was assessed using FastQC (Version 0.10.1). Adapters, low-quality reads at the 3′ end, reads with fuzzy N bases, ribosomal RNA (rRNA), sequences shorter than 20 nt and low-quality reads (those with a Q < 20) were trimmed with the FASTX clipper (Version 0.0.13). All the double-end reads in eight samples from two treatment groups were separately aligned to the chicken reference genome (Gallus_gallus-5.0, version 81, Ensembl) using the spliced mapping algorithm in TopHat2 (version: 2.0.9) (37). Unless stated otherwise, all programs were run with the default parameters. The number of reads equivalent to mapped reads [reads per kilobase per million mapped reads (RPKM)] was used to normalize the expression of each gene. The quality of the obtained data was assessed based on the presence and abundance of contaminating sequences, the average read length, and the GC content. With the exception of the microarray design, the NGS experiment conformed to the MIAME guidelines (38).

### Bioinformatics Analysis

All data processing steps for differential gene expression evaluation were performed using the Cuffdiff software package (DNASTAR, Madison, Wisconsin, USA). Further analysis was conducted only using genes that demonstrated a >1.5-fold change or a <0.7-fold change in expression between the groups, as demonstrated by *t*-tests with an adjusted *P*-value ≤ 0.05. A Benjamini and Hochberg test (with an error rate of 0.05) and a false discovery rate correction test (FDR < 0.05) were used to adjust the *P*-values. The biological mechanisms underlying the DEGs were investigated using DAVID v. 6.7 software (the Database for Annotation, Visualization, and Integrated Discovery) (39) (http://david.abcc.ncifcrf.gov/). The sets of genes were uploaded using official gene IDs. Finally, the molecular interaction networks of the DEGs were investigated using OmicsBean (http://www.omicsbean.cn) and Cytoscape v. 3.1.0 software (http://www.cytoscape.org/) (40) as a complementary and more comprehensive approach for identifying the central hub genes. The results were visualized using the ClueGO v. 2.1.1. application in Cytoscape, which creates clusters of functionally related genes using the following GO databases: KEGG, Wiki Pathways, REACTOME, and GO.

### LncRNA Identification

To identify novel reliable lncRNA models, we considered only multiexonic transcripts and filtered them through the following highly stringent criteria: (1) size selection: only transcripts with ≥200 bp were kept; (2) read coverage threshold: transcripts with ≤ 3 reads were removed from our dataset; (3) open reading frame (ORF) filter: transcripts with a predicted ORF longer than 100 aa were removed; (4) known protein domain filter: transcripts were aligned to the Pfam and Swiss-Protein databases to eliminate transcripts with significant homology to known protein domains (41); and (5) protein-coding-score test: both the Coding-Non-Coding Index (CNCI) and the CPC were used to evaluate the coding potential of the candidate lncRNAs (42, 43). Further analysis was conducted only with the lncRNAs that showed >1-log2 (fold change) or <-1-log2 (fold change) differential expression between the dietary groups, as demonstrated through *t*-tests with an adjusted *P*-value ≤ 0.05.

### RNA Fluorescence *in situ* Hybridization (RNA FISH)

Cy3-labeled lncRNA- XLOC_098131 probes were obtained from RiboBio (Guangzhou, China). RNA FISH was performed using a fluorescent *in situ* hybridization kit (RiboBio) following the manufacturer's instructions.

### 5′- and 3′-Rapid Amplification of cDNA Ends (RACE)

To determine the full-length sequence of lncRNA- XLOC_098131, RACE experiments were performed using the SMARTer RACE cDNA Amplification Kit (Clontech, Palo Alto, California, USA) following the manufacturer's recommended protocol. The gene-specific primers used for 5′ and 3′ RACE were GTTCTGCAGGAGAGCAGCAG and AAGTGGCCTGAGCTGGAGTC, respectively.

### Constructs

The full length of lncRNA- XLOC_098131 contains a 3′UTR luciferase reporter WT plasmid named pHS-AVC-LW406 (psi-SV40 promoter-hRluc- lncRNA- XLOC_098131-HSV TK promoter-hluc) (Figures S1A,B); a mutant expression plasmid of lncRNA- XLOC_098131 without the predicted miR-548s-binding sites named pHS-AVC-LW407 [psi-SV40 promoter-hRluc- lncRNA- XLOC_098131 (mutation)-HSV TK promoter-hluc] (Figures S1C,D), mir-548s overexpression plasmids named pHS-AMR-LW010 (pZDonor_hef1a-EYFP-T2A-puro-hsa-mir-548ah) (Figure S1E), pHS-AMR-LW011 (pZDonor_hef1a-EYFP-T2A-puro-hsa-mir-548ay) (Figure S1F), and pHS-AMR-LW012 (pZDonor_hef1a-EYFP-T2A-puro-hsa-mir-548e) (Figure S1G), and a control mi plasmid named pHS-AMR-LW013 (pZDonor_hef1a-EYFP-T2A-puro-control miRNA) (Figure S1H). These plasmids were constructed by Hesheng Gene Company (Beijing, China) to test the ability of lncRNA- XLOC_098131 to bind to mir-548s. The full length of FOS contains a 3′UTR luciferase reporter WT plasmid named pHS-AVC-LW480 [psi-SV40 promoter-hRluc-Fos gene part (hsa-miRNA-548ay-3p target)-HSV TK promoter-hluc] (Figure S2A) and a mutant expression plasmid of FOS without the predicted miR-548s-binding sites named pHS-AVC-LW481 [psi-SV40 promoter-hRluc-Fos gene part mutation (hsa-miRNA-548ay-3p target mutation)-HSV TK promoter-hluc] (Figure S2B), and these were constructed by Hesheng Gene Company (Beijing, China) to test the binding between FOS and mir-548s. An FOS luciferase vector named pHS-AVC-LW482 (psi-SV40 promoter-hRluc-Fos gene part (hsa-miRNA-548ay-3p target)-HSV TK promoter-hluc) (Figure S2C), an lncRNA- XLOC_098131 overexpression plasmid and a mutant overexpression plasmid of lncRNA- XLOC_098131 without predicted miR-548s-binding sites (Figure S2D) were constructed by Hesheng GenePharma Company (Beijing, China) to verify that lncRNA- XLOC_098131 regulates FOS expression by competitively binding to miR-548s. We also constructed a mutant expression plasmid of lncRNA- XLOC_098131 without predicted miR-1180s-binding sites named pHS-AVC-LW251 [psi-SV40 promoter-hRluc- lncRNA- XLOC_098131 (mutant)-HSV TK promoter-hluc] (Figure S3B) and mir-1180s overexpression plasmids named pHS-AMR-ZQ011 (pZDonor_hef1a-EYFP-T2A-puro-hsa-mir-1180-5P) and pHS-AMR-ZQ012 (pZDonor_hef1a-EYFP-T2A-puro-hsa-mir-1180-3P) (Figure S3A) to test the binding between lncRNA- XLOC_098131 and mir-1180s.

### Luciferase Assays

For the luciferase reporter assays, HEK293FT cells were seeded into 96-well plates in triplicate and transfected with the WT lncRNA- XLOC_098131 construct pHS-AVC-LW406, the mutant lncRNA- XLOC_098131 construct pHS-AVC-LW407, psiCHECK-2 with the mir-548s overexpression plasmids pHS-AMR-LW010, pHS-AMR-LW011, and pHS-AMR-LW012, or the control miR plasmid pHS-AMR-LW013. Forty-eight hours after transfection, the luciferase activities were measured using a Dual-Luciferase® Reporter Assay System (Promega). HEK293FT cells were then seeded into 96-well plates in triplicate and transfected with the WT FOS construct pHS-AVC-LW480, the mutant FOS construct pHS-AVC-LW481, psiCHECK-2 with the mir-548s overexpression plasmids pHS-AMR-LW010, pHS-AMR-LW011, and pHS-AMR-LW012, or the control mi plasmid pHS-AMR-LW013. Forty-eight hours after transfection, the luciferase activities were measured. Additionally, HEK293FT cells were seeded into 96-well plates in triplicate and transfected with the FOS luciferase vector pHS-AVC-LW480 or control psiCHECK with pcDNA3.1, lncRNA- XLOC_098131, lncRNA- XLOC_098131-mut or lncRNA- XLOC_098131+mir-548s. Forty-eight hours after transfection, the luciferase activities were measured. In addition, the luciferase assays of JUN and lncRNA- XLOC_098131 were the same as those used in the FOS assay.

### Quantitative Real-Time Reverse Transcription PCR

The expression levels of various genes were analyzed by qRT-PCR. The gene-specific primer sequences are shown in Table 2, and the analysis was performed on a 7500-fluorescence detection system (Applied Biosystems, Foster City, California, USA) using a commercial SYBR-Green PCR kit (Takara Bio Inc. Foster City, California, USA). According to the manufacturer's recommended protocol, the following PCR conditions were employed: 95°C for 30 s and 40 cycles of 95°C for 5 s and 60°C for 34 s. In addition, melting curve analyses and subsequent agarose gel electrophoresis of the PCR products were conducted to confirm the amplification specificity. The relative gene expression data were analyzed using the 2^−ΔΔCt^ method.

**Table 2:** qRT-PCR primers for gene analysis.

**Target**	**Primer sequence (5**′**-3**′**)^a^**	**Product size, bp**
FOS	F: CGGGAGAGGAACAAGATGG R: TCCTTCAGCAGGTTGGCTAT	137
CTSK	F: AGTCTGCCCTCCTTCCAGTT R: TGCTTGGTGCCCTTCTGT	119
BDKRB1	F: GTGTATCGACGCCATCTGTG R: GACAGCCAGGTTCACAAGGT	118
KL	F: TCCAGGAACGACCAAGAAGT R: CAACGCTGTTTCTCTGGTGA	130
CYR61	F: CTGCAGAGCACAGTCTGAGG R: CAGCCCACAGCTCCATCTAT	120
CDH2	F: GCCCATTGACTTTGAGACCA R: GACACGGTTGCTGTTGACTG	111

a *F refers to forward, and R refers to reverse*.

### Statistical Analysis

When a significant difference was observed between treatments, the individual treatment means were compared with Duncan's multiple comparison using SPSS Version 18.0. A *P*-value < 0.05 was considered to indicate statistical significance, and *P* < 0.1 was considered to indicate a trend toward statistical significance.

## Results

### Serum Immunoglobulin Levels, Peripheral Blood Lymphocyte Classification, and Lymphocyte Proliferation in Hens

**Figure 1:**
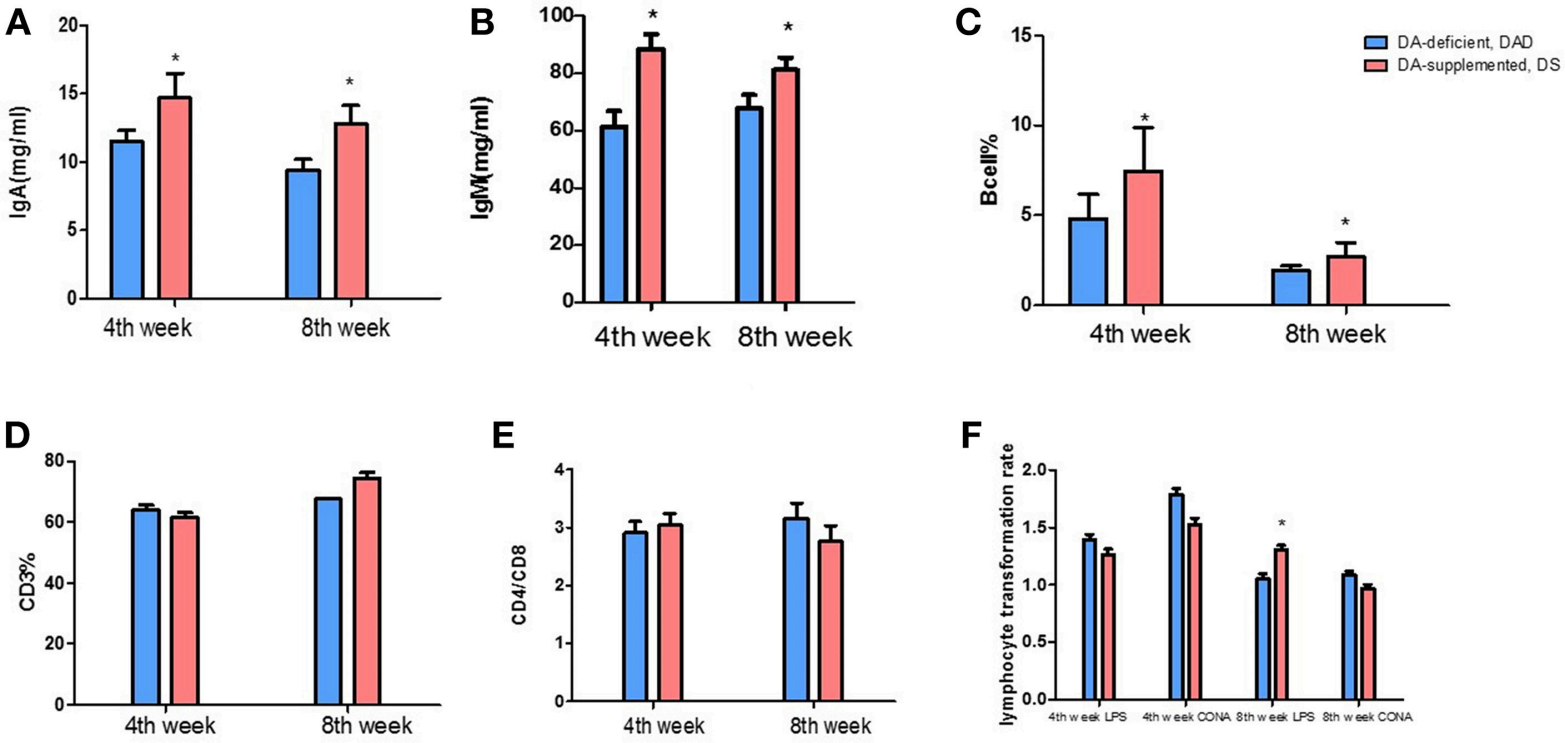
Effects of dietary daidzein supplementation of hens on the serum immunoglobulin levels and peripheral blood lymphocyte classification and lymphocyte proliferation. **(A)** Serum immunoglobulin A levels of hens at the 4 and 8th weeks of the experiment. **(B)** Serum immunoglobulin M levels of hens at the 4 and 8th weeks of the experiment. **(C)** Peripheral blood B lymphocyte percentages of hens at the 4 and 8th weeks of the experiment. **(D)** Peripheral blood CD3(+) T cell percentages of hens at the 4 and 8th weeks of the experiment. **(E)** Peripheral blood CD4/CD8 T cells of hens at the 4 and 8th weeks of the experiment. **(F)** *In vitro* lymphocyte proliferation in hens at the 4 and 8th weeks of the experiment. Data information: We induced T and B lymphocyte cell multiplication in peripheral blood lymphocytes through concanavalin A and lipopolysaccharide stimulation, respectively. LPS, lipopolysaccharide stimulus index; CON A, concanavalin A stimulus index. The results are expressed as the means ± SEMs. Bars labeled with “*” are significantly different (*P* < 0.05).

Compared with the DA-deficient (DAD) group, DA supplementation at a dose of 20 mg/kg in hens increased (*P* < 0.05) the serum IgA and IgM levels on the 4 and 8th weeks of the experiment, as shown in Figures 1A,B. The effects of DA treatment on peripheral blood lymphocytes are also shown in Figures 1C–F. DA supplementation at a dose of 20 mg/kg increased (*P* < 0.05) the ratio of B cells on the 4 and 8th weeks of the experiment (Figure 1C), but no significant effect (*P* > 0.05) on the percentage of CD3 (Figure 1D) and the CD4/CD8 ratio was observed (Figure 1E). We further examined the lipopolysaccharide (LPS)- and concanavalin A (CON A)-mediated stimulation of the lymphocyte proliferation rate and found that the DA treatment increased (*P* < 0.05) the proliferative responses of B cells on the 8th week of the experiment, as shown by changes in the LPS stimulation index (SI) values obtained *in vitro* using peripheral blood (Figure 1F). In contrast, no difference in lymphocyte proliferation was observed following CON A stimulation.

### Routine Blood Examination

The routine blood examination results (Table 3) showed that on the 4th week of the experiment, the platelet count (PLT) and pressure (PCT) in the DA-supplemented (DS) group were decreased (*P* < 0.05) compared with those found in the DAD group, and the DS group also exhibited a slight increase (*P* < 0.1) in the neutrophil ratio (NEUT%). Furthermore, DA significantly increased the percentage of monocytes (MONO%) and the proportion of large unstained cells (LUC%). On the 8th week of the experiment, the MONO and LUC% in the DS group were higher (*P* < 0.05) than those in the DAD group, and the NEUT% showed an increasing tendency (*P* < 0.1). In addition, the DA treatment did not affect the white blood cell percentage (WBCP), average platelet volume (MPV), platelet distribution width (PDW), lymphocyte ratio (lymph%), eosinophilia ratio (EOS%), or basophil ratio (BASO%).

**Table 3:** Effects of dietary daidzein supplementation of hens on routine blood examination results^*^.

	**WBCP**	**PLT**	**MPV**	**PDW**	**PCT**	**NEUT(%)**	**Lymph(%)**	**MONO(%)**	**EOS(%)**	**LUC(%)**	**BASO(%)**
**Week 4**											
DAD	76.29	55.38^a^	41.85	23.65	0.23^a^	14.23	79.58	4.34^b^	0.19	0.66^b^	0.63
DS	76.10	36.38^b^	41.71	24.83	0.15^b^	19.99	80.68	6.71^a^	0.23	1.11^a^	0.46
SEM	3.634	3.617	0.298	0.785	0.016	1.618	1.502	0.554	0.027	0.102	0.060
*P*-value	0.980	0.004	0.827	0.474	0.006	0.073	0.728	0.026	0.499	0.022	0.183
**Week 8**											
DAD	79.74	53.13	41.88	21.54	0.25	15.37	73.14	4.49^b^	0.26	1.09^b^	0.76
DS	83.47	69.38	36.41	23.05	0.24	20.65	70.33	7.29^a^	0.31	1.73^a^	0.63
SEM	4.585	6.772	1.794	0.689	0.037	1.577	2.182	0.555	0.033	0.155	0.088
*P-*value	0.699	0.243	0.132	0.295	0.847	0.095	0.538	0.006	0.464	0.034	0.455

*WBCP, white blood cell percentage (%); PLT, platelet count (10^9^/L); MPV, mean platelet volume (fL); PDW, platelet volume distribution width (%); PCT, platelet thrombocytocrit (%); NEUT (%), neutrophil ratio (%); lymph%, lymphocyte ratio (%); MONO%, monocyte ratio (%); EOS%, eosinophil ratio (%); LUC%, proportion of large unstained cells (%); BASO%, basophil ratio (%). The data are expressed as the means with SEMs and P-values*.

* *Values with the same superscript within a column are not significantly different at P < 0.05, values with the different superscript (a,b) are significantly different at P < 0.05*.

### RNA-Seq Statistics

**Figure 2:**
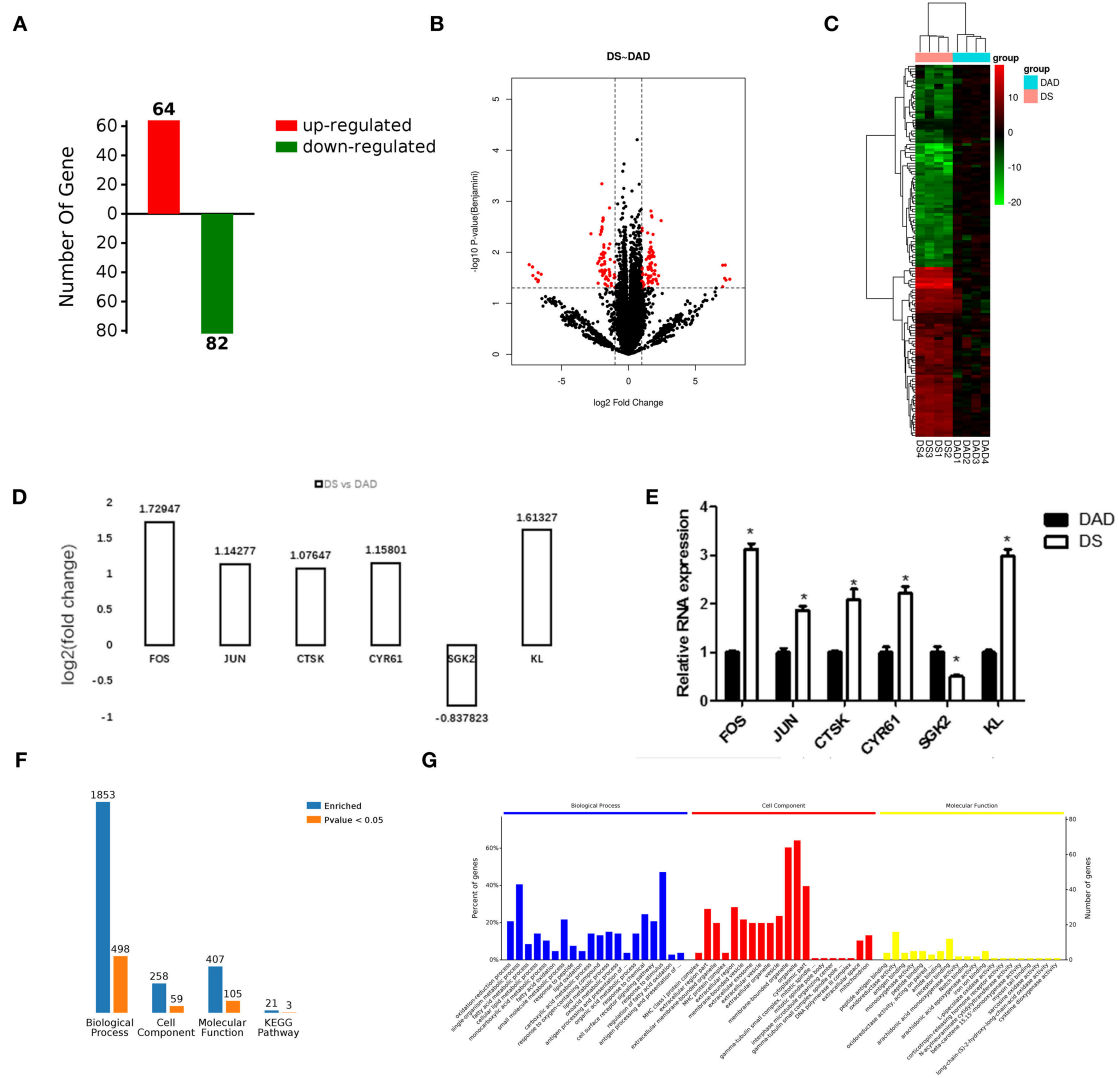
Effects of dietary daidzein supplementation of hens on differential gene expression and functional enrichment. **(A)** Number of differentially expressed genes (DEGs) (up/downregulated) in hens after daidzein supplementation at 20 mg/kg. **(B)** Heatmap of expression of DEGs in samples. DAD, daidzein-deficient group; DS, daidzein-supplemented group. DAD group samples: DAD1-4; DS group samples: DS1-4. **(C)** Volcano map of the expression of DEGs. **(D)** Fold change based on transcriptome results for six DEGs. **(E)** Relative RNA expression of the six genes by qRT-PCR. **(F)** Number of DEGs enriched in GOs and pathways. **(G)** Top 20 significantly enriched biological processes, cell components, and molecular functions.

In this study, we established eight cDNA libraries from the livers of the birds in the DAD and DS groups with four replicates per group. The RNA-Seq assay generated 79,846,624–93,885,866 raw reads per library, with an average of 88,374,633 and 83,729,690 paired-end reads in the DAD and DS groups, respectively. After filtering the low-quality reads, the average numbers of clean reads in the DAD and DS groups were 82,831,728 (93.73%) and 74,604,252 (89.10%), respectively. The clean reads were used in all further analyses. Approximately 83.0% of the reads in each library were uniquely mapped to the galGal4 assembly of the chicken genome, and the average mapping rates of the DAD and DS groups were 92.7 and 93%, respectively (Table S1). As shown in Figure 2A, 146 differentially expressed genes (DEGs; 64 upregulated and 82 downregulated) were identified in the comparison of the DAD group with the DS group (FDR ≤ 0.05, fold change ≥1.5 or ≤ 0.7) using Cuffdiff software. The expression levels and fold changes of the DEGs are shown in Table S2, and the DEGs are also shown in a heat map (Figure 2B) and volcano plot (Figure 2C). Moreover, the transcriptome data of the DEGs were validated by RT-PCR (Figure 2E), and the results were consistent with the RNA-Seq results (Figure 2D).

### Functional Categorization of DEGs and Pathway Analysis

We performed a functional enrichment analysis using the 146 DEGs, and results showed revealed 1,853, 259, 407, and 21 terms associated with biological process regulation, cell components, molecular function, and Kyoto Encyclopedia of Genes and Genomes (KEGG) pathways, respectively, and 498, 59, 105, and 3 significantly enriched terms in these categories, respectively (Figure 2F). The DA treatment mainly affected the GO terms: oxidation-reduction process, fatty acid metabolic process, response to peptides, response to oxygen-containing compounds, antigen processing, presentation of peptide antigens via MHC class I and other biological processes; MHC class I protein complex, extracellular region portion, extracellular membrane-bound organelle, extracellular region, membrane-bound vesicle, and other cell components; and peptide antigen binding, oxidoreductase activity, antigen binding, receptor binding, and other molecular functions (Figure 2G). A complete list of the different gene ontology (GO) terms identified in this analysis is provided in Table S3.

**Figure 3:**
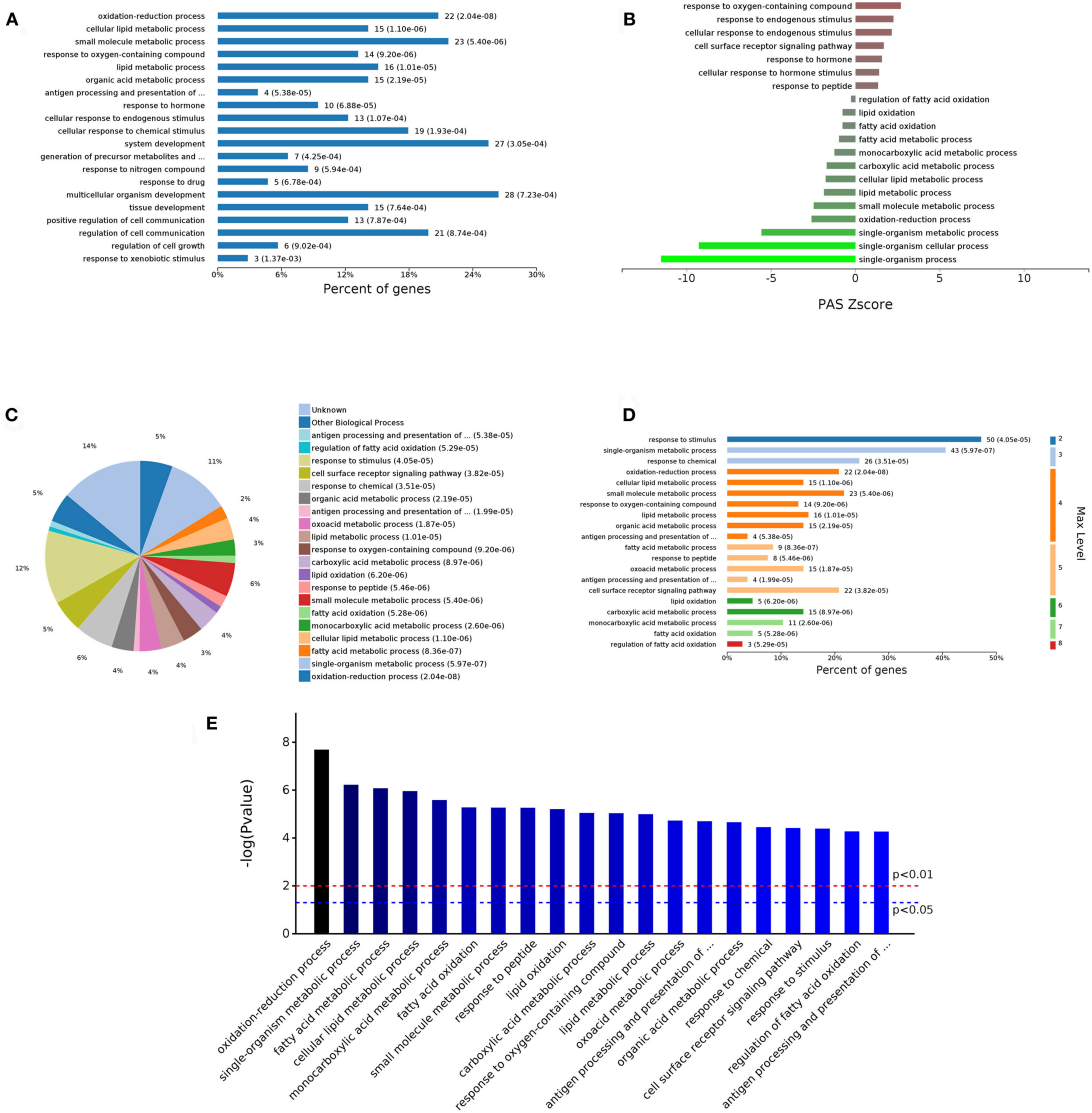
Analysis of 498 different biological process GOs. **(A)** Percent of genes associated with enriched biological processes. **(B)** PAS value of enriched biological processes. **(C)** Expressed proteins associated with enriched biological process. **(D)** Levels of enriched biological processes. **(E)** Significantly enriched biological processes.

The 498 different biological process GO terms were further analyzed using OmicsBean software. According to the number of enriched genes (Figures 3A,C), the DEGs identified from the comparison of the DS group with the DAD group were mainly enriched in multicellular organism development (28 genes), system development (27 genes), and oxidation-reduction process (22 genes). According to the different levels of the GO terms (Figure 3D), most differential genes were related to stimuli (level 2 GO), and continued subdivision of the grades showed that the differential genes were involved in the organism response to multiple stimuli (level 3–8 GO). A sorting of the different GO terms based on the *P*-value (Figure 3E) revealed that these DEGs are most likely involved in biological processes related to antioxidation and the immune response. The PAS *Z*-score (Figure 3B) combines the *P*-value and fold change data and showed that compared with the DAD group, the DS group had significantly more immune response-related GO terms associated with enhanced immunity and significantly fewer terms related to lipid oxidation and other processes, which indicates that DS can be associated with an enhancement of the body's antioxidant capacity.

**Figure 4:**
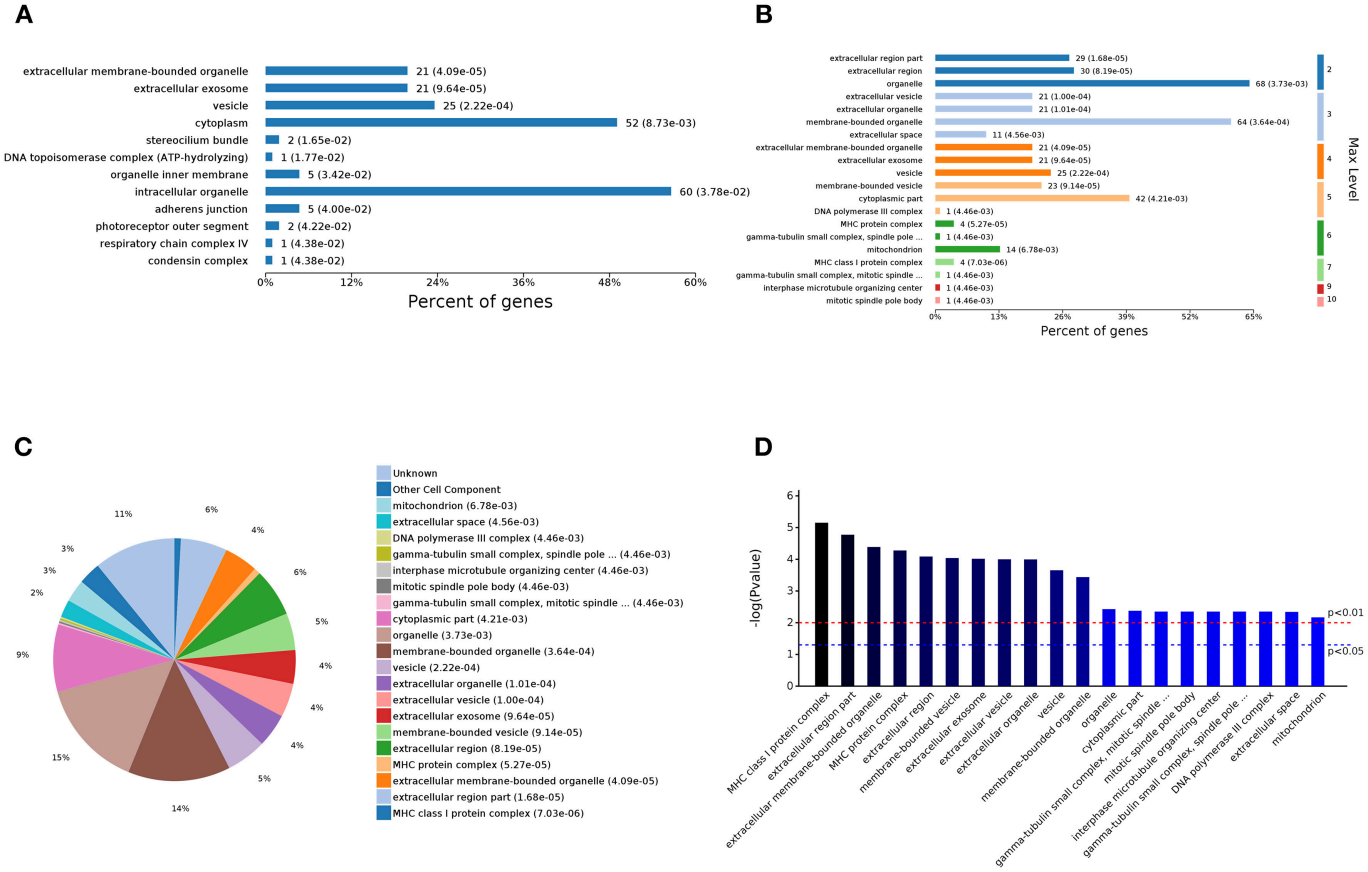
Analysis of 59 different cell component GOs. **(A)** Percent of genes associated with enriched cell components. **(B)** Levels of enriched cell components. **(C)** Expressed proteins associated with enriched cell components. **(D)** Significantly enriched cell components.

The analysis of the 59 different cell components according to the number of enriched genes (Figures 4A,C) revealed that the most abundant DEGs were enriched in intracellular organelles (60 genes) and cytoplasm (52 genes). In terms of the different GO classification levels (Figure 4B), the genes showing the highest differential expression are associated with organelles (level 2 GO), and further subdivisions showed that the DEGs are associated with membrane-bound organelles (level 3 GO). Sorting the different GO terms based on ascending *P*-values (Figure 4D) revealed that the genes showing the greatest differential expression are likely enriched in the cellular component of the MHC protein complex.

**Figure 5:**
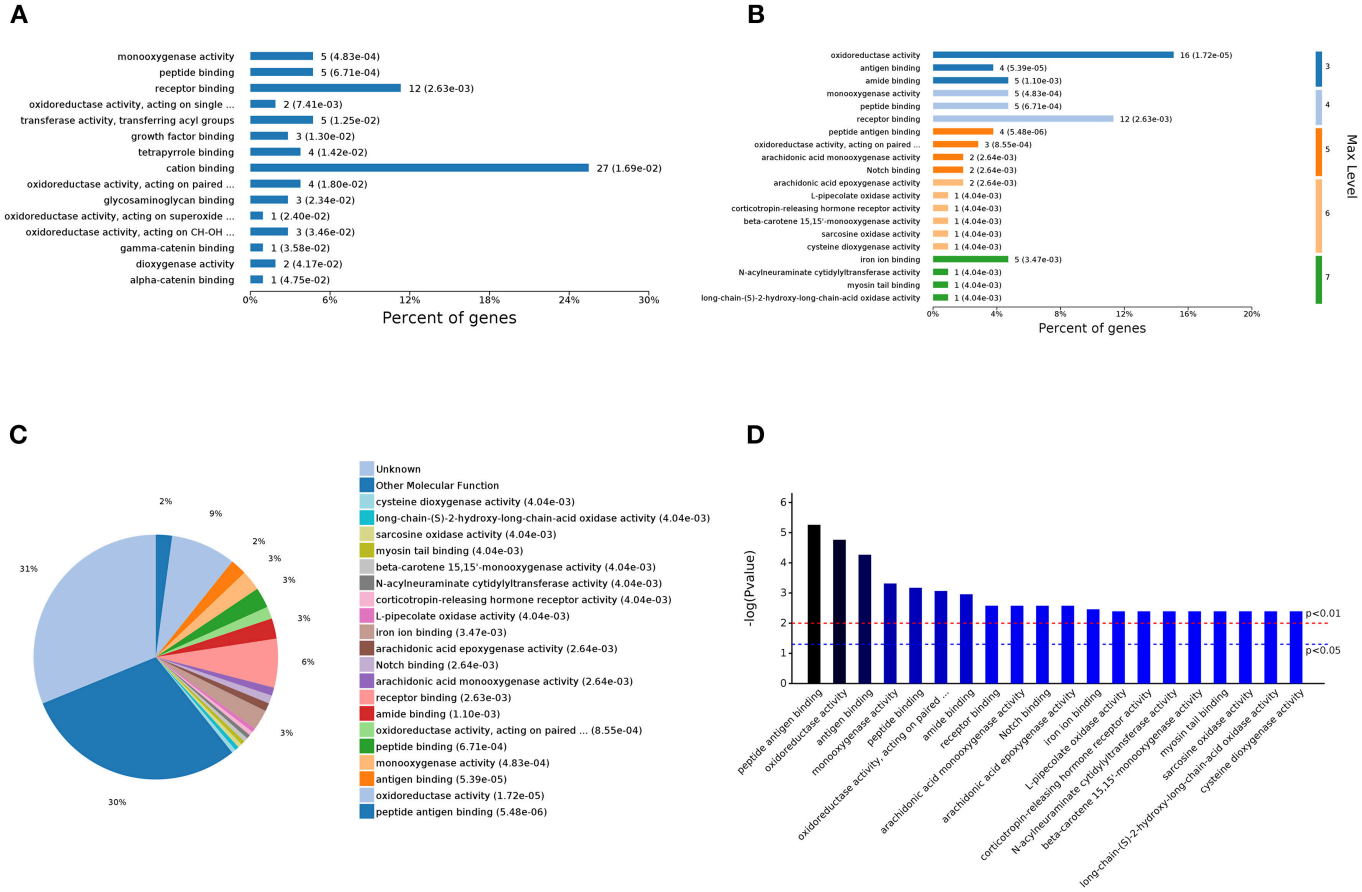
Analysis of 105 different molecular function GOs. **(A)** Percent of genes associated with enriched molecular functions. **(B)** Levels of enriched molecular functions. **(C)** Expressed proteins associated with enriched molecular functions. **(D)** Significantly enriched molecular functions.

The analysis of the 105 different molecular function-related GO terms showed that according to the number of enriched genes (Figures 5A,C), the most abundantly enriched DEGs are associated with cation binding (27 genes) and receptor binding (12 genes). According to the classification of the different GO levels (Figure 5B), most DEGs are related to continued subdivision identified receptor binding (level 4 GO) and peptide antigen binding (level 5 GO). By distinguishing the difference in the *P*-value of the GO terms (Figure 5D), we found that the molecular functions of these DEGs are peptide antigen binding and oxidoreductase activity.

Based on the above results, it can be hypothesized that the DA treatment had an impact on the immune response, and a statistical analysis of immune regulation-related GO terms (Table 4) showed that the differential genes are involved in the positive regulation of responses to stimulus, interleukin-8 endocytosis involved viral entry into host cell, chemical stimulus, drug stimulus, endogenous stimulus, abiotic stimulus, oxidative stress, reactive oxygen species, and other immune responses. The following immunity-related GO terms were also regulated: negative regulation of B cell apoptotic processes, antigen binding, interleukin-1-mediated signaling pathway, transport of virus, regulation of MAPK cascade, positive regulation of MAP kinase activity, regulation of MAP kinase activity, Toll-like receptor 21 signaling pathway activation of MAPK activity involved in innate immune response, and receptor-mediated endocytosis of the virus by the host cell.

**Table 4:** Effects of dietary daidzein supplementation of hens on DEGs used in GO clustering analysis related to the immune system.

**Gonane**	**GO_ID**	***P*-value**	**Genes|Fold change**	**Count**
Response to interleukin-8	GO:0098758	6.95E-03	EGR1|2.376	1
Endocytosis involved in viral entry into host cell	GO:0075509	1.04E-02	CAV2|0.417	1
Cellular response to endogenous stimulus	GO:0071495	1.71E-05	CAV2|0.417;ACSL1|0.614;CYR61|2.231;EGR1|2.376;JUN| 2.208;RARA|1.526;FOS|3.316;CCK1R|5.905;CHRDL1| 0.429;IRS2|1.866;KL|3.059;CRHR2|2.007;PDK4|0.421	13
Cellular response to peptide hormone stimulus	GO:0071375	3.94E-04	CAV2|0.417;IRS2|1.866;KL|3.059;CRHR2|2.007;PDK4|0.421	5
Cellular response to chemical stimulus	GO:0070887	1.81E-03	CAV2|0.417;SOD3|0.184;ACSL1|0.614;CYR61|2.231;EGR1| 2.376;JUN|2.208;PPARGC1A|0.564;RARA|1.526;FOS| 3.316;CCK1R|5.905;CHRDL1|0.429;IRS2|1.866;KL|3.059;CRHR2| 2.007;PDK4|0.421	15
Interleukin-1-mediated signaling pathway	GO:0070498	4.77E-02	EGR1|2.376	1
Response to stimulus	GO:0050896	9.99E-04	NIM1K|1.617;CAV2|0.417;CDH2|1.906;SOD3|0.184;SLC25A25| 0.627;CRCP|0.599;ACSL1|0.614;MST1R|1.705;GFRA3| 0.646;CHAC1|0.521;ENPP2|0.6;EVC|0.468;CYR61|2.231;EGR1| 2.376;JUN|2.208;PPARGC1A|0.564;TOP2A|0.53;RARA|1.526;FOS| 3.316;IGFBP1|0.446;WFDC1|0.417;GPX2|7.618;SIK1| 1.694;CCK1R|5.905;BCO1|0.596;KIAA1324|5.521;CHRDL1| 0.429;SGK2|0.559;IRS2|1.866;KL|3.059;LPAR3| 2.868;SLC6A14|0.423;CRHR2|2.007;TRPA1| 0.123;ANKRD6|0.513;EVC2|0.376;DUSP8|1.567;JAG1| 1.703;MCC|2.125;PDK4|0.421	40
Positive regulation of response to stimulus	GO:0048584	3.07E-03	CAV2|0.417;CDH2|1.906;MST1R|1.705;EVC| 0.468;CYR61|2.231;JUN|2.208;KIAA1324|5.521;KL| 3.059;LPAR3|2.868;CRHR2|2.007;ANKRD6|0.513;JAG1| 1.703	12
Regulation of response to stimulus	GO:0048583	2.59E-03	CAV2|0.417;CDH2|1.906;MST1R|1.705;CHAC1| 0.521;EVC|0.468;CYR61|2.231;EGR1| 2.376;JUN|2.208;IGFBP1|0.446;WFDC1| 0.417;KIAA1324|5.521;CHRDL1| 0.429;KL|3.059;LPAR3|2.868;CRHR2|2.007;ANKRD6| 0.513;DUSP8|1.567;JAG1|1.703;MCC|2.125	19
Transport of virus	GO:0046794	3.76E-02	CAV2|0.417	1
Regulation of MAPK cascade	GO:0043408	8.82E-05	CAV2|0.417;CDH2|1.906;MST1R|1.705;CYR61|2.231;JUN| 2.208;KL|3.059;LPAR3|2.868;ANKRD6|0.513;DUSP8|1.567	9
Positive regulation of MAP kinase activity	GO:0043406	2.76E-02	MST1R|1.705;JUN|2.208;LPAR3|2.868	3
Regulation of MAP kinase activity	GO:0043405	1.10E-02	MST1R|1.705;JUN|2.208;LPAR3|2.868;DUSP8|1.567	4
Response to drug	GO:0042493	2.12E-02	JUN|2.208;FOS|3.316;TRPA1|0.123	3
Toll-like receptor 21 signaling pathway	GO:0035682	4.10E-02	JUN|2.208	1
Activation of MAPK activity involved in innate immune response	GO:0035419	4.43E-02	JUN|2.208	1
Cellular response to reactive oxygen species	GO:0034614	3.40E-02	SOD3|0.184;FOS|3.316	2
Cellular response to oxidative stress	GO:0034599	1.58E-02	SOD3|0.184;PPARGC1A|0.564;FOS|3.316	3
Cellular response to hormone stimulus	GO:0032870	2.94E-05	CAV2|0.417;ACSL1|0.614;JUN|2.208;RARA| 1.526;CCK1R|5.905;IRS2|1.866;KL|3.059;CRHR2| 2.007;PDK4|0.421	9
Receptor-mediated endocytosis of virus by the host cell	GO:0019065	1.04E-02	CAV2|0.417	1
Response to endogenous stimulus	GO:0009719	1.66E-05	CAV2|0.417;ACSL1|0.614;CYR61|2.231;EGR1| 2.376;JUN|2.208;RARA|1.526;FOS| 3.316;CCK1R|5.905;CHRDL1|0.429;IRS2| 1.866;KL|3.059;CRHR2|2.007;JAG1| 1.703;PDK4|0.421	14
Response to abiotic stimulus	GO:0009628	2.83E-02	SOD3|0.184;EGR1|2.376;JUN|2.208;FOS|3.316;SIK1|1.694;TRPA1|0.123	6
Response to mechanical stimulus	GO:0009612	6.05E-03	JUN|2.208;FOS|3.316;TRPA1|0.123	3
Response to external stimulus	GO:0009605	1.69E-03	SLC25A25|0.627;MST1R|1.705;GFRA3|0.646;ENPP2| 0.6;JUN|2.208;PPARGC1A|0.564;FOS| 3.316;WFDC1|0.417;SIK1|1.694;KIAA1324| 5.521;CRHR2|2.007;TRPA1| 0.123;PDK4|0.421	13
Response to oxidative stress	GO:0006979	2.44E-03	SOD3|0.184;PPARGC1A|0.564;FOS|3.316;GPX2|7.618;TRPA1|0.123	5
Antigen binding	GO:0003823	5.39E-05	LOC100859408|0.197785696238; ENSGALG00000004772|0.374843424568; LOC768350|0.376374293512; ENSGALG00000027944|0.511967097185	4
Negative regulation of B cell apoptotic process	GO:0002903	2.75E-02	IRS2|1.866	1
Response to reactive oxygen species	GO:0000302	8.32E-03	SOD3|0.184;FOS|3.316;TRPA1|0.123	3

**Figure 6:**
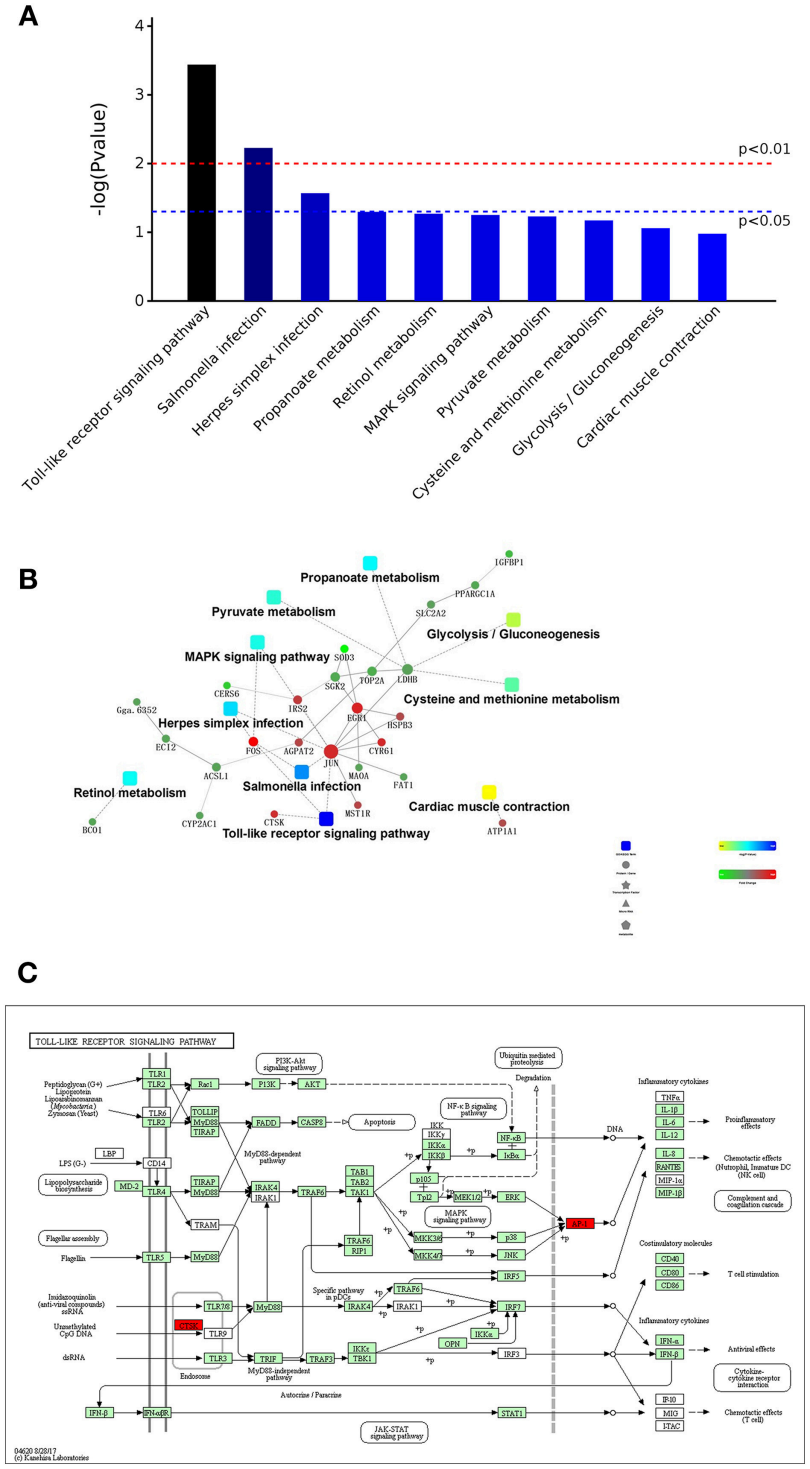
KEGG pathway cluster analysis and correlation analysis of DEGs. **(A)** Distribution of enriched KEGG pathways. **(B)** Correlation analysis of DEGs. **(C)** Daidzein treatment regulated the Toll-like signaling pathway. The key factors are marked in red.

Through a KEGG pathway cluster analysis, we also found that the DEGs identified from the comparison of the DAD group with the DS group regulated the Toll-like receptor signaling pathway, salmonella infection pathway and herpes simplex infection pathway (Figure 6A). A correlation analysis (Figure 6B) showed that the DEGs are mainly involved in the Toll-like receptor signaling pathway, salmonella infection, herpes simplex infection, MAPK signaling pathway, pyruvate metabolism, cysteine and methionine metabolism, and cardiac muscle contraction, and the key genes regulating these processes are JUN, FOS, EGR1, IRS2, CTSK, and CYR61. Furthermore, an analysis of the most significant Toll-like receptor signaling pathway in KEGG (Figure 6C) revealed that the CTSK (upstream) gene in the pathway and the dimer AP-1, which is composed of proteins encoded by the JUN and FOS (downstream) genes in the pathway, play an important role in DA-mediated regulation of immunity. AP-1 can respond to a variety of stimuli, including cytokines, growth factors, stress, and bacterial and viral infections, by regulating the expression of downstream genes. This finding is consistent with the results of the GO analysis, which showed that DA addition mainly enhanced the body's ability to respond to multiple stimuli. Therefore, FOS and JUN are key genes that enable the DA-mediated enhancement of the immune response and innate immunity in hens.

### Differential lncRNA Selection

**Figure 7:**
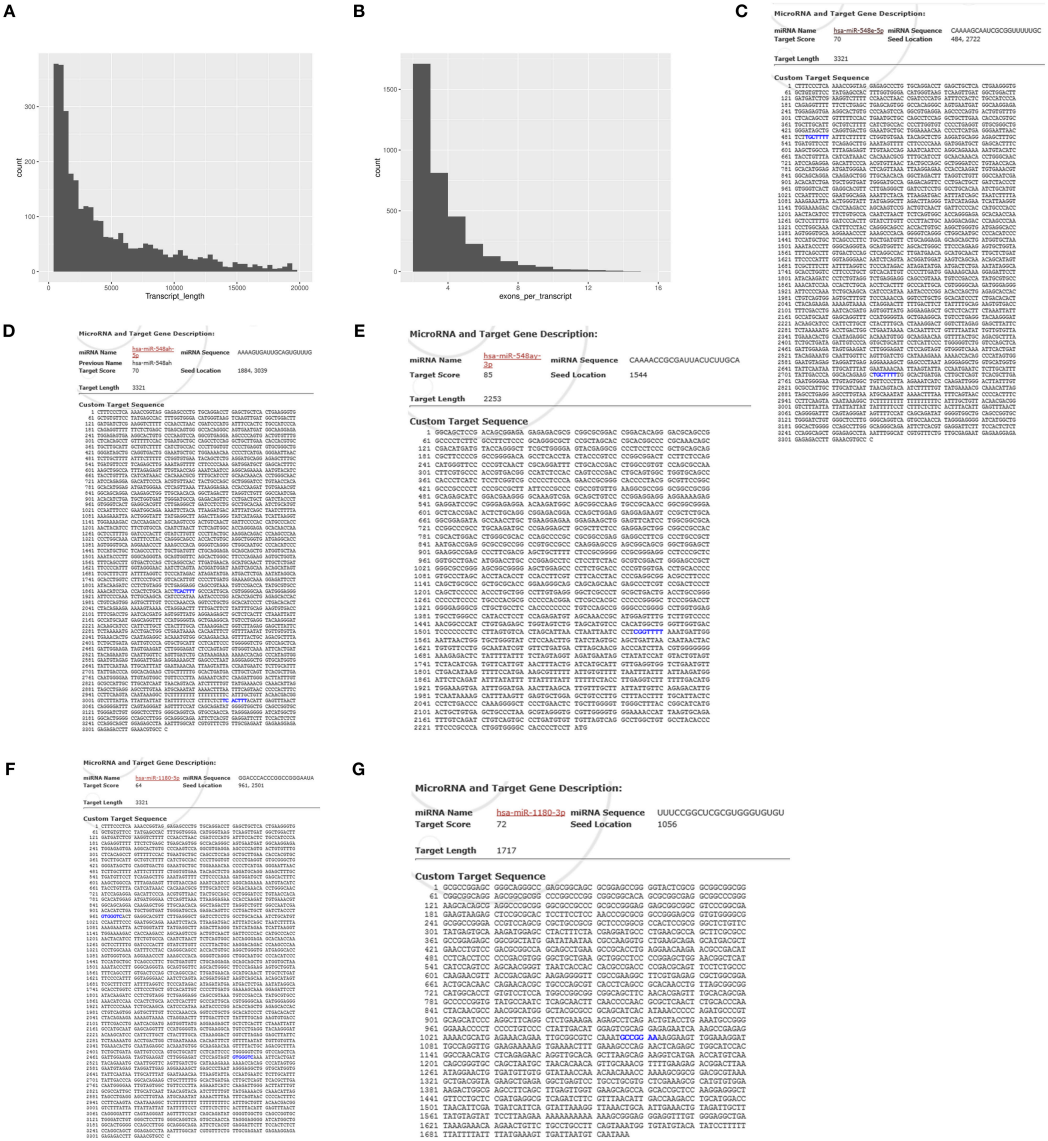
Transcript length and transcript exons of lncRNAs and predicted binding sites for gene sequences and miRNAs. **(A)** Transcript length of lncRNAs. **(B)** Exon numbers of lncRNA transcripts. **(C)** Predicted binding sites of lncRNA- XLOC_098131 and miR-548s (miR-548e-5p). **(D)** Predicted binding sites of lncRNA- XLOC_098131 and miR-548s (miR-548 ah−5p). **(E)** Predicted binding sites of FOS and miR-548s (miR-548ay-3p). **(F)** Predicted binding sites of lncRNA- XLOC_098131 and miR-1180s. **(G)** Predicted binding sites of JUN and miR-1180s.

We performed a lncRNA screen of the DS and DAD groups and identified 1,095 lncRNAs (Table S4). An analysis of the transcript lengths and transcript exons of all lncRNAs (Figures 7A,B) revealed that the frequency of these lncRNA transcripts was mainly concentrated in 200–5,000 units and that the number of transcript exons mainly ranged from 2 to 4. LncRNAs that demonstrated differential expression >1-log2 (fold change) or <-1-log2 (fold change) between the two groups, as determined through *t*-tests with an adjusted *P*-value ≤ 0.05, were selected. In the DS group, nine and 22 lncRNAs were upregulated and downregulated, respectively compared with their levels in the DAD group (Table 5).

**Table 5:** Differentially expressed lncRNAs between the daidzein-supplemented group and the daidzein-deficient group.

**Gene_id**	**Locus**	**log2(fold_change)**	**q_value**
XLOC_098131	8:25819102-25826005	2.25348	0.002783
XLOC_053963	20:5515116-5515913	2.49987	0.004555
XLOC_101028	9:17660403-17663648	1.95707	0.010438
XLOC_108821	JH375740.1:3495-5053	2.92619	0.010736
XLOC_110897	Z:13526884-13531884	1.64843	0.012525
XLOC_106453	AADN03020969.1:48-1144	1.98815	0.021471
XLOC_060507	3:4438760-4440121	2.22644	0.02505
XLOC_107859	AADN03026913.1:0-1654	1.40834	0.029407
XLOC_060807	3:44996672-45004147	1.27564	0.045252
XLOC_107884	AADN03027032.1:5-1157	−4.00884	0.002783
XLOC_106739	AADN03022135.1:0-1077	−3.96327	0.002865
XLOC_106700	AADN03021946.1:8-1557	−3.12889	0.002987
XLOC_107647	AADN03026044.1:748-1538	−3.03662	0.003083
XLOC_105225	AADN03015717.1:329-1427	−2.81266	0.003313
XLOC_107752	AADN03026468.1:23-1490	−2.25076	0.003465
XLOC_106646	AADN03021718.1:202-1080	−2.00632	0.002567
XLOC_105024	AADN03014889.1:21-1062	−1.63664	0.002783
XLOC_105701	AADN03017712.1:5-1287	−1.80265	0.004555
XLOC_031304	14:13653237-13661582	−1.5396	0.010736
XLOC_109853	JH376401.1:5284-11263	−1.54183	0.012525
XLOC_109110	JH376055.1:0-1722	−1.40485	0.014735
XLOC_106264	AADN03020084.1:8-995	−2.81169	0.020875
XLOC_109206	JH376151.1:21542-23697	−1.84632	0.021095
XLOC_107372	AADN03024860.1:0-1023	−2.79506	0.021293
XLOC_105135	AADN03015347.1:7-363	−1.42235	0.037575
XLOC_105285	AADN03015983.1:0-2072	−1.44937	0.043086
XLOC_106988	AADN03023146.1:0-1058	−4.39913	0.043356
XLOC_107076	AADN03023608.1:86-1023	−1.37271	0.045232
XLOC_104526	AADN03012670.1:70-1718	−1.30948	0.042342
XLOC_107295	AADN03024536.1:0-2094	−1.11588	0.045252
XLOC_109383	JH376260.1:1543-5603	−1.08636	0.045456

### Cis-Regulation Analysis of Differential lncRNAs

A screen of all mRNAs within 10 kb from the 31 differentially expressed lncRNAs yielded 392 mRNAs (Table S5). A comparing of these 392 mRNAs with transcriptome mRNA expression data did not result in a significant difference in the expression of these 392 mRNAs between the DS and DAD groups, suggesting that the differentially expressed lncRNAs identified from the comparison of the DAD and DS groups cannot regulate the expression of adjacent mRNAs through cis-regulation.

### Potential Binding Sites in the lncRNAs and the mRNAs

To explore the mechanism underlying the action of the differentially expressed lncRNAs and identify the targets, we analyzed the potential binding sites in the lncRNAs and the mRNAs of FOS and JUN, which are key genes that enable the DA-mediated enhancement in the immune response, using mirDB (http://www.mirdb.org/miRDB/). The results showed that lncRNA- XLOC_098131 has two sites for binding to miR-548s (miR-548e-5p), and the seed locations are 484 and 2,722 (Figure 7C). In addition, lncRNA- XLOC_098131 has two binding sites for miR-548s (miR-548ah-5p), and the seed locations are 1,884 and 3,039 (Figure 7D). Furthermore, FOS has a binding site for miR-548s (miR-548ay-3p) located at 1,544 (Figure 7E), and lncRNA- XLOC_098131 has two binding sites for miR-1180s, with seed locations of 961 and 2,501 (Figure 7F). Moreover, JUN has a binding site for miR-1180s located at 1,506 (Figure 7G). These results suggest that lncRNA- XLOC_098131 might regulate the expression of FOS and JUN by competitively binding to miR-548s and miR-1180s.

### Characterization of the lncRNA- XLOC_098131 Sequence

**Figure 8:**
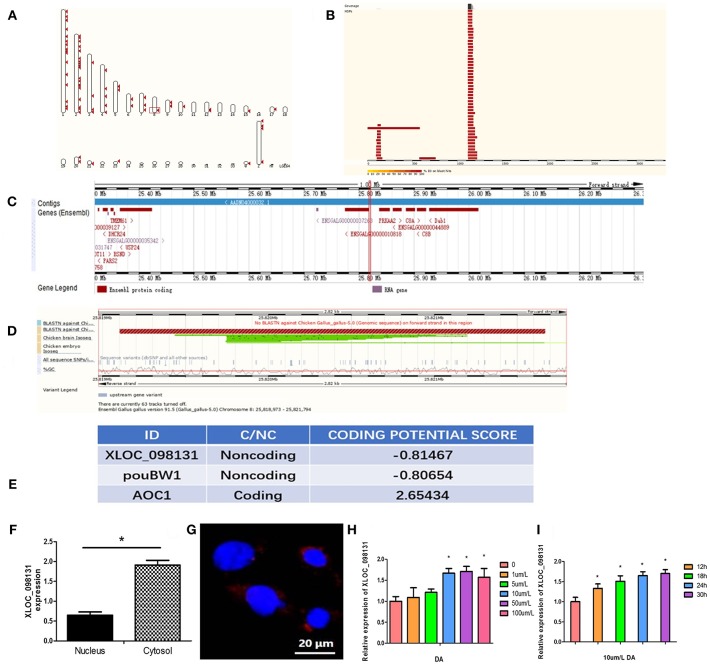
Characterization of the lncRNA-XLOC_098131 sequence. **(A)** High-scoring segment pair (HSP) is a local alignment with no gaps that achieves one of the highest alignment scores in a given search. It corresponds to the matching region between the query and the database hit sequence. **(B)** The HSP distribution can be visualized on the query, which is shown as a chain of black and white boxes. Fragments of the query sequence that hit other places in the genome are shown as red boxes. **(C)** Detailed overview of the region. The gene colors are as follows: gold and red genes: protein coding; gray, blue, and purple genes: non-coding; the red squares indicate the location of lncRNA- XLOC_098131. **(D)** BLASTN against Chicken Gallus_gallus-5.0 (Genomic sequence), Chicken brain Iso-Seq, and Chicken embryo Iso-Seq. Sequence variants (dbSNP and all other sources): sequence variants from all sources; %GC: percentages of G and C bases in the region. **(E)** CPC (Coding Potential Calculator) values predicted for lncRNA- XLOC_098131, pouBW1, and AOC1. **(F)** Expression of lncRNA- XLOC_098131 transcripts in the nuclear and cytosolic of chicken hepatocyte cells. **(G)** LncRNA- XLOC_098131 FISH of chicken hepatocyte cells. LncRNA- XLOC_098131 is shown in red, and the color blue indicates the cell nucleus. **(H)** Relative expression of lncRNA- XLOC_098131 in primary chicken hepatocytes after treatment with different doses of daidzein. **(I)** Relative expression of lncRNA- XLOC_098131 in primary chicken hepatocytes after treatment with daidzein for various times.

The 5′ and 3′ rapid amplification of cDNA ends (RACE) analyses demonstrated that lncRNA- XLOC_098131 is a transcript consisting of 3,321 nucleotides, and similar to many lncRNAs, this transcript is polyadenylated. The full length of lncRNA- XLOC_098131 is shown in Table S6. The full length of lncRNA- XLOC_098131 was then used in a BLASTN search with the genomic sequences of chicken, human, mouse, pig, cow, and sheep using the online tool Ensemble (http://asia.ensembl.org/) and considering an e-val<0.05. This search yielded more than 100 chicken alignment sequences, one human alignment sequence, one mouse alignment sequence, seven pig alignment sequences, one cow alignment sequence, and four sheep alignment sequences. The results of the first ten comparisons between chickens and other animals are shown in Table 6. For the chicken alignment, the sequence at genomic location 8:25819102–25821666 scored 5,085 with an e-val of 0; the sequence at genomic location 8:25825425–25826005 scored 1,152 with an e-val of 0; and the sequence at genomic location 8:25822459–25822638 scored 357 with an e-val of 1.00E-95. These results are consistent with the transcriptome results, which suggests that lncRNA- XLOC_098131 is located in 8:25819102–25826005. Furthermore, analyses of the high-scoring segment pair (HSP) distribution on the genome (Figure 8A) and HSP distribution on the query sequence (Figure 8B) revealed that lncRNA- XLOC_098131 is located on chromosome 8 in chicken.

**Table 6:** BLASTN analysis of the lncRNA- XLOC_098131 sequence with the genomic sequence of multiple species.

**Species**	**Genomic location**	**Overlapping gene(s)**	**Orientation**	**Query start**	**Query end**	**Length**	**Score**	**E-val**	**%ID**
Chicken (Gallus gallus)	8:25819102-25821666		Reverse	757	3,321	2,565	5,085	0	100
	8:25825425-25826005		Reverse	1	581	581	1,152	0	100
	8:25822459-25822638		Reverse	580	759	180	357	1.00E-95	100
	6:10061666-10061723	PRKG1	Reverse	1,122	1,179	58	83.8	2.00E-13	93.1
	2:102877343-102877419		Forward	1,122	1,199	78	83.8	2.00E-13	89.74
	1:13229048-13229148	RELN	Reverse	1,122	1,222	102	83.8	2.00E-13	87.25
	1:188522666-188522723		Forward	1,122	1,179	58	83.8	2.00E-13	93.1
	2:148971284-148971360	ENSGALG00000046235	Forward	1,123	1,199	77	81.8	1.00E-12	88.31
	2:60329403-60329449		Forward	100	146	47	77.8	2.00E-11	95.74
	2:146079337-146079383		Forward	1,122	1,168	47	77.8	2.00E-11	95.74
Human (Homo sapiens)	16:84600302-84600329		Forward	2,952	2,979	28	56	0.002	100
Mouse (Mus musculus)	11:88368731-88368764	Msi2	Reverse	3,005	3,038	34	60	2.00E-05	97.06
Pig (Sus scrofa)	10:12597715-12597743	NVL	Forward	2,963	2,991	29	50.1	0.007	96.55
	8:75192172-75192196		Reverse	2,952	2,976	25	50.1	0.007	100
	2:5886714-5886738		Forward	2,966	2,990	25	50.1	0.007	100
	9:131770635-131770666		Forward	2,963	2,994	32	48.1	0.028	93.75
	6:40493835-40493866	ZNF536	Forward	2,963	2,994	32	48.1	0.028	93.75
	5:67610724-67610755		Reverse	2,963	2,994	32	48.1	0.028	93.75
	1:91969563-91969590		Forward	486	513	28	48.1	0.028	96.43
Cow (Bos taurus)	18:4693512-4693539	ADAMTS18	Reverse	2,963	2,990	28	48.1	0.03	96.43
Sheep (Ovis aries)	13:76463289-76463318	PREX1	Reverse	2,950	2,979	30	52	0.002	96.67
	1:142389415-142389447		Reverse	3,008	3,040	33	50.1	0.007	93.94
	3:180456894-180456925		Reverse	2,963	2,994	32	48.1	0.029	93.75
	26:36177735-36177762		Forward	2,952	2,979	28	48.1	0.029	96.43

*Genomic location, location of the hit on the genome; Overlapping gene(s), genes that overlap the genomic location hit; Orientation, strand of the genome the query sequence is found on; Query start and end, positions within the query sequence where the alignment begins and ends (1-based, inclusive); Length, full length of the alignment, including all gaps in either the query or the alignment; Score, BLAST score calculated from the alignment; E-val, probability that the alignment between the query sequence and subject sequence is due to chance; %ID, percentage of the aligned query sequence that is identical to the subject sequence*.

As shown in Figure 8C, lncRNA- XLOC_098131 is located on chromosome 8 at position 25819102–25821666 (illustrated by the red line), which is a non-coding area between the two protein-coding genes ENSGALG00000010818 and protein kinase AMP-activated catalytic subunit alpha 2 (PRKAA2) and near the ENSGALG00000044889 gene, which encodes complement C8 alpha chain (C8A), C8B, and DAB1. We performed a further BLASTN analysis, as shown in Figure 8D, and the BLASTN against Chicken Gallus_gallus-5.0 (Genomic sequence) showed no BLASTN pairing with lncRNA- XLOC_098131 and a forward strand orientation in the chicken genome sequence, but BLASTN revealed a high degree of matching with the reverse direction of the chicken genome sequence (lncRNA- XLOC_098131 is a reverse sequence). Furthermore, the BLASTN analysis of lncRNA- XLOC_098131 with the chicken brain Iso-Seq from the European Nucleotide Archive (https://www.ebi.ac.uk/ena) (44) resulted in a high degree of matching, which was slightly lower than that obtained with Chicken Gallus_gallus-5.0 (Genomic sequence); thus, the tissue specificity of lncRNA- XLOC_098131 is low in chickens. The BLASTN analysis of lncRNA- XLOC_098131 with chicken embryo Iso-Seq from the European Nucleotide Archive (https://www.ebi.ac.uk/ena) (44) resulted in a lower degree of matching, showing that the expression level of lncRNA- XLOC_098131 gradually increases with chicken development and highlighting the high temporal specificity of lncRNA- XLOC_098131. The analysis of lncRNA- XLOC_098131 sequence variants from all sources showed that the variability in the sequence of lncRNA- XLOC_098131 is low, indicating that lncRNA- XLOC_098131 has high sequence stability. Furthermore, the percentages of bases G and C in the sequence (%GC) are high, indicating that the sequence of lncRNA- XLOC_098131 has high stability. In addition, consistent with the fact that lncRNA- XLOC_098131 is a non-coding RNA, the Coding Potential Calculator (CPC) computational algorithm predicted that lncRNA- XLOC_098131 has a very low coding potential, similar to pouBW1, which is a well-documented Gallus lncRNA (45) (Figure 8E). These results indicate that lncRNA- XLOC_098131 is a real long non-coding reverse RNA sequence with good stability and low specificity between tissues but with a high temporal specificity and a certain degree of conservation among species.

### Cellular Localization of lncRNA- XLOC_098131

To determine the cellular localization of the lncRNA- XLOC_098131 transcript, the nuclear, and cytosolic RNAs from chicken primary hepatocytes were isolated, and the expression of lncRNA- XLOC_098131 transcripts in both subcellular locations was measured. The qRT-PCR data showed that lncRNA- XLOC_098131 transcripts were more highly expressed in the cytosol than in the nucleus (Figure 8F). A FISH analysis of lncRNA- XLOC_098131 in hepatocytes also indicated that lncRNA- XLOC_098131 is mainly located in the cytoplasm (Figure 8G).

### DA Treatment Can Upregulate the Expression of lncRNA- XLOC_098131 *in vitro*

Chicken primary hepatocytes were treated with 0, 1, 5, 10, 50, or 100 μm/L DA for 24 h, and the total RNA from the cells was then extracted for RT-PCR. The 10, 50, and 100 μm/L DA treatment groups exhibited significantly increased expression of lncRNA- XLOC_098131 compared with the control group (Figure 8H). We then examined cultured cells treated with 10 μm/L DA for 0, 12, 18, 24, and 30 h and found that the cells treated with 10 μm/L DA for 12, 18, 24, or 30 h exhibited significantly increased expression of lncRNA- XLOC_098131 compared with the control group (Figure 8I).

### Binding Between lncRNA- XLOC_098131 and miR-548s

**Figure 9:**
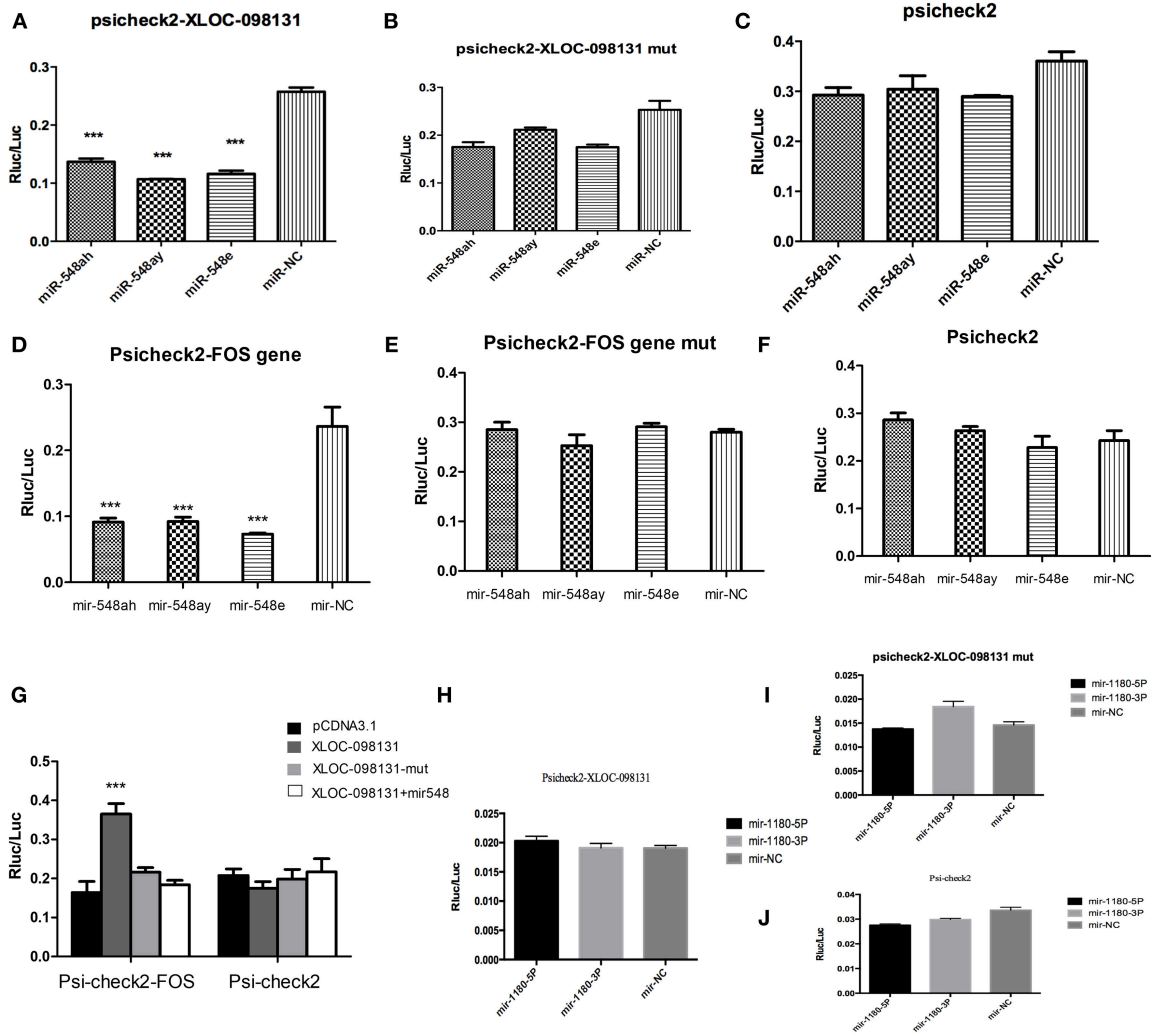
Characterization of the lncRNA-XLOC_098131 sequence. **(A)** Luciferase activity in HEK293FT cells cotransfected with miR-548s and lncRNA-XLOC-098131. **(B)** Luciferase activity in HEK293FT cells cotransfected with miR-548s and lncRNA-XLOC-098131 mutant transcript. **(C)** Luciferase activity in HEK293FT cells cotransfected with miR-548s and luciferase reporters containing nothing. **(D)** Luciferase activity in HEK293FT cells cotransfected with miR-548s and FOS. **(E)** Luciferase activity in HEK293FT cells cotransfected with miR-548s and FOS mutant transcript. **(F)** Luciferase activity in HEK293FT cells cotransfected with miR-548s and luciferase reporters containing nothing. **(G)** Luciferase activity in HEK293FT cells cotransfected with FOS and lncRNA- XLOC_098131, lncRNA- XLOC_098131-mut, lncRNA- XLOC_098131+miR-548s or pCDNA3.1. Luciferase activity in HEK293FT cells cotransfected with Psi-check2 and lncRNA- XLOC_098131, lncRNA- XLOC_098131-mut, lncRNA- XLOC_098131+miR-548s or pCDNA3.1. **(H)** Luciferase activity in HEK293FT cells cotransfected with miR-1180s and lncRNA-XLOC-098131. **(I)** Luciferase activity in HEK293FT cells cotransfected with miR-1180s and lncRNA-XLOC-098131 mutant transcript. **(J)** Luciferase activity in HEK293FT cells cotransfected with miR-1180s and luciferase reporters containing nothing. Data information: The data are presented as the relative ratios of Renilla luciferase activity to firefly luciferase activity.

To assess the existence of direct binding between miR-548s and lncRNA- XLOC_098131 at endogenous levels, we constructed luciferase reporters containing lncRNA- XLOC_098131 that contained wild-type (WT) or mutated miR-548s-binding sites. We found that the overexpression of miR-548s reduced the luciferase activities of the WT reporter vector (Figure 9A) but not that of the empty vector (Figure 9C) or that of the mutant reporter vector (Figure 9B). These data demonstrated that the predicted miR-548s could regulate luciferase expression with the 3′UTR of lncRNA- XLOC_098131 (*P* < 0.05). Mutation of the binding site resulted in the disappearance of this regulation, confirming that miR-548s regulates luciferase through this binding site and that lncRNA- XLOC_098131 might be a target gene of miR-548s.

### Binding Between FOS and miR-548s

To validate the hypothesized direct binding between miR-548s and FOS at endogenous levels, we constructed luciferase reporters containing FOS that contained WT or mutated miR-548s-binding sites. We found that miR-548s overexpression reduced the luciferase activities of the WT reporter vector (Figure 9D) but not that of the empty vector (Figure 9F) or that of mutant reporter vector (Figure 9E). These data demonstrated that the predicted miR-548s could regulate luciferase expression with the 3′UTR of FOS (*P* < 0.05). This regulation disappeared if the binding site was mutated, confirming that miR-548s regulates luciferase through this binding site and that FOS might be a target gene of miR-548s.

### LncRNA- XLOC_098131 Upregulates FOS Expression by Serving as a Competitive Endogenous RNA

Because lncRNA- XLOC_098131 shares the regulatory miR-548s with FOS, we questioned whether lncRNA- XLOC_098131 could modulate FOS by regulating miR-548s. As shown in Figure 9G, the overexpression of lncRNA- XLOC_098131 WT, but not that of the mutant or lncRNA- XLOC_098131, increased the FOS transcript level. In addition, the co-transfection of the lncRNA- XLOC_098131 WT overexpression vector, FOS luciferase vector, and sufficient miR-548s resulted in no difference in the luciferase activity of the FOS luciferase vector compared with the control group, and the above treatments had no effect on the psiCHECK-2 luciferase activity.

### Binding Between lncRNA- XLOC_098131 and miR-1180s

To validate the existence of direct binding between miR-1180s and lncRNA- XLOC_098131 at endogenous levels, we constructed luciferase reporters containing lncRNA- XLOC_098131 that contained WT or mutated miR-1180s-binding sites. However, compared with the control, the overexpression of miR-1180s could not reduce the luciferase activities of the WT reporter vector (Figure 9H), the mutant reporter vector (Figure 9I) or the empty vector (Figure 9J). This result showed that lncRNA- XLOC_098131 cannot bind to miR-1180s through our predicted binding site. The regulation of the JUN gene by DA treatment is not achieved through the competitive regulation of lncRNA- XLOC_098131, and thus, the mechanism underlying the regulation of JUN expression by DA treatment needs further investigation.

## Discussion

Immunoglobulins (Ig), which are key factors in the immune response, prevent infection, can facilitate lysis and activate complements to promote phagocytosis. Previous studies have shown that treating bulls with 100, 200, or 400 ppm DA for 60 days increased their IgA and IgM levels (46), and DA can increase the bovine serum IgE and IgM levels (47). Our results demonstrated that DA supplementation at a dose of 20 mg/kg increased the serum IgA and IgM levels in hens on the 4 and 8th weeks of the experiment, which is consistent with previous research. The peripheral blood lymphocyte content and proliferation ability are important indicators of the body's immune response, and appropriately high levels of lymphocytes in the peripheral blood and a better ability to respond to stimuli are important manifestations of a good immune response (48, 49). Studies investigating lymphocytes following DA treatment have shown that the ability of offspring broilers' B, but not T, lymphocytes to differentiate might be enhanced by adding DA to the breeder's diet at a dose of 20 mg/kg (35). In our study, 20 mg/kg DA increased the number of B lymphocytes in the peripheral blood in hens but had no significant effect on CD3(+) subset of T lymphocytes or on the CD4(+)/CD8(+) T cell ratio. Furthermore, the DA treatment resulted in an increase in the proliferative responses of B cells, as shown by the changes in the LPS SI values obtained *in vitro* using peripheral blood from hens on the 8th week of the experiment, but had no effect on the proliferative responses of T cells, which is consistent with the previous finding that 0.5 mg/kg DA has no significant effect on T lymphocyte proliferative responses in 5- to 6-week-old boars (50). These results suggest that the effect of DA on lymphocytes is mainly manifested in the regulation of B lymphocytes and that the effect on T lymphocytes is markedly lower. Enhanced B lymphocyte differentiation produces more plasma cells that can secrete more immunoglobulins, which is consistent with the finding that the DA treatment increased the serum IgA and IgM levels in hens. The transcriptome results showed that compared with the DAD group, the DS group exhibited significantly upregulated IRS2 gene expression, which is associated with the inhibition of B lymphocyte apoptosis. Additionally, DA treatment can promote IgA and IgM secretion by regulating the expression of antigen-presenting related genes, such as LOC100859408, ENSGALG00000004772, and LOC768350. Previous studies have found that soy isoflavones, including DA, can regulate the expression of the IRS2 gene, which might affect B lymphocytes (51). Therefore, treatment with 20 mg/kg DA can reduce B lymphocyte apoptosis, promote the differentiation of B lymphocytes into plasma cells that, in turn, secrete more immunoglobulins, promote the presentation of antigens and improve humoral immunity in hens.

Properly elevated levels of neutrophils and mononuclear cells in the blood can increase the body's ability to respond to external stimuli and bacterial infections and enhance immunity (52, 53). Based on our results, the DS group tended to exhibit an increase in the number of neutrophils in the blood of hens on the 4 and 8th weeks of the experiment (*P* < 0.1), and the number of monocytes in the DS group was significantly higher than that in the DAD group. The RNA-Seq results showed that the DEGs identified from the comparison of two groups such as EGRI1, CAV2, JUN, FOS, SOD3, and CDH2 are involved in the regulation of the interleukin-8 endocytosis viral entry into the host cell, the regulation of Toll-like receptor 21 signaling pathway and MAPK signaling pathway, which can modulate the neutrophils and macrophages. These results show that DA treatment can enhance the body's resistance to viruses and harmful bacteria, enhance the immune response to various stimuli, and improve the body's innate immunity. Previous studies have demonstrated that DA can regulate the content of monocytes and neutrophils and relieve toxic symptoms in mice (54) and that DA can play a positive role in regulating LPS-induced lung injury by regulating the neutrophil content (55).

The KEGG and correlation analyses performed in this study showed that the DS group had an enhanced ability to respond to multiple stimuli compared with the DAD group, and this enhanced ability was regulated by the MAPK signaling pathway, Toll-like pathway and salmonella-related pathway, which can regulate downstream genes to improve humoral and innate immunity, as discussed above. We found that the key genes regulating these processes are JUN, FOS, EGR1, IRS2, CTSK, and CYR61, and the dimer AP-1 composed of JUN and FOS plays a crucial regulatory role in the pathway. AP-1 can regulate the expression of multiple genes to respond to multiple stimuli, including cytokines, growth factors, stress, and bacterial and viral infections. Some studies have found that an appropriately high expression of AP-1 can enhance the body's immune and antibacterial ability and improve its ability to respond to multiple stimuli (56). The AP-1 family of transcription factors can activate Toll-like receptor agonists and positively regulate interleukin-4 (IL-4) to activate macrophages, thereby enhancing antibacterial activity (57). AP-1 plays a key role in regulating immunity as an intermediate regulatory factor; for example, CR3 and Dectin-1 synthesize macrophage cytokines through synergistic activation of the lipid and Syk-JNK-AP-1 pathways (58). Previous studies have shown that multiple plant estrogens can activate AP-1 expression (59), and the treatment of cells with DA significantly increases their expression of the FOS gene (60). DA can also increase FOS gene and protein expression in humans (61). Therefore, in our study, FOS and JUN were identified as the key genes controlling the immunity of hens following DA treatment. The addition of 20 mg/kg DA to the breeder hen diet can upregulate the expression of FOS, JUN, and AP-1 to regulate the Toll-like receptor signaling pathway, MAPK pathway, salmonella infection signal pathway, and related mRNA expression to enhance macrophage activity and increase blood neutrophils and mononuclear cells. This process improves the body's ability to respond to cytokines, growth factors, stress, and bacterial and viral infections and improves the innate immunity.

This study explored the role of lncRNAs in the regulation of key immunity-related genes following DA treatment. We sequenced lncRNAs that showed differential expression between the DA treatment and control groups, identified 1,096 lncRNAs, and found that the frequency of these lncRNA transcripts was mainly concentrated in the 200–5,000 unit range and that the number of transcript exons ranged from 2 to 4, which is consistent with the characteristics of lncRNAs(6). Furthermore, nine and 22 lncRNAs were significantly upregulated and downregulated after DA treatment. We further analyzed the relationship between the differentially expressed lncRNAs and key mRNAs. Previous studies have shown that lncRNAs can influence the expression of adjacent mRNAs that are located within 10 kb of the lncRNAs through cis-regulatory effects; for example, the lncRNA Malat1 plays a cis-regulatory role in the adult mouse (62), and the lncRNA GAPLINC regulates mRNA by cis-regulation in gastric cancer (63). In this study, we selected 31 differentially expressed lncRNAs for the analysis and found that 392 mRNAs are located near these lncRNAs. However, none of these mRNAs exhibited significantly differential expression between the DAD and DS groups, suggesting that the DA treatment could not regulate the expression of mRNAs by the cis-regulatory function of the lncRNAs that were found to be differentially expressed between the two groups. Many studies have found that lncRNAs function as competitive endogenous RNAs to regulate mRNA expression. For example, the lncRNA CCAT1 upregulates the proliferation and invasion of melanoma cells by inhibiting miR-33a (64), the lncRNA XIST regulates AKT expression by competitively binding miR-494 (65), and the lncRNA TUG1 binds to microRNA-9 to regulate the expression of Bcl2l11 (66). By predicting binding sites using an online tool, we found that lncRNA- XLOC_098131, which is the most highly upregulated lncRNA after DA treatment, might regulate FOS expression by competitively binding to miR-548s and regulate JUN expression by competitively binding to miR-1180s. Therefore, lncRNA- XLOC_098131 was selected for further experimentation. First, we also verified that DA treatment can upregulate lncRNA- XLOC_098131 expression *in vitro*. We then found that lncRNA- XLOC_098131 is a novel transcript located on chromosome 8 in chickens between 25819102 and 25826005, and a BLASTN analysis showed that this fragment is located in a non-coding sequence region, indicating that lncRNA- XLOC_098131 is a non-coding transcript. For further confirmation, we used the CPC computational algorithm and predicted that lncRNA- XLOC_098131 has a very low coding potential, similarly to pouBW1, a well-documented Gallus lncRNA. As several studies have revealed, lncRNAs have low tissue specificity in animals (67), and we found that a BLASTN analysis of lncRNA- XLOC_098131 with chicken brain Iso-Seq resulted in a high degree of matching, which was slightly lower than that obtained from the BLASTN analysis with the chicken genomic sequence. These findings demonstrate the low tissue specificity of lncRNA- XLOC_098131. However, further analysis revealed that lncRNA- XLOC_098131 has low expression in the chicken embryo and that its expression gradually increases with development, highlighting the high temporal specificity of lncRNA- XLOC_098131, and these results are consistent with previous research (68). LncRNAs are known to have high stability and a certain degree of conservation (69). We found that the variability of the sequence of lncRNA- XLOC_098131 is low and that the percentages of the bases G and C in the sequence (%GC) are high, indicating that the sequence of lncRNA- XLOC_098131 exhibits high stability. These results indicate that lncRNA- XLOC_098131 is a real long non-coding reverse RNA sequence with good stability and low specificity between tissues but with a high temporal specificity and a certain degree of conservation among species. LncRNAs can be distributed in the nucleus or cytoplasm, and lncRNAs in the cytoplasm can regulate the expression of mRNAs as ceRNAs (70). In this study, we conducted PCR and FISH experiments to show that lncRNA- XLOC_098131 is mainly located in the cytoplasm, where lncRNAs can bind to miRNAs through their binding sites and thereby eliminate the miRNA-mediated inhibition of mRNAs, which can also bind to miRNAs (71). We successfully demonstrated that both lncRNA- XLOC_098131 and FOS are target genes of miR-548s and that lncRNA- XLOC_098131 can bind to mir-548s through its binding sites to reduce the level of free miR-548s, which in turn reduces the binding of FOS and miR-548s, eliminating the miR-548s-mediated inhibition of FOS and upregulating the expression of FOS. Furthermore, when found at a sufficient high level, the binding of miR-548s to lncRNA- XLOC_098131 did not affect the intracellular free mir-548s content; thus, there was no change in the number of miR-548s that bound to FOS, and the expression of FOS was unchanged. These results confirmed that lncRNA- XLOC_098131 regulates FOS expression by competitively binding to miR-548s. However, subsequent experiments demonstrated that lncRNA- XLOC_098131 cannot regulate JUN gene expression by competitively binding to miR-1180s. LncRNAs can also affect mRNA expression through epigenetic or transcriptional regulation (6), and the molecular mechanism through which lncRNAs regulate JUN expression needs further investigation.

In summary, our study demonstrates that a novel lncRNA activated by DA can stabilize FOS mRNA by serving as a competitive endogenous RNA and can affect AP-1 with JUN to regulate the MAPK signaling, Toll-like receptor signaling, and salmonella signaling pathways *in vivo*. Dietary supplement DA can improve the numbers of blood neutrophils and mononuclear cells, and improve the innate immunity response. In addition, by increasing the content and differential ability of B lymphocytes and improving antigen presentation and immunoglobulin secretion, DA enhances the body's humoral immunity. In conclusion, our study shows that the novel lncRNA- XLOC_098131 plays a key role in the molecular mechanism underlying the immune function mediated by DA. This study provides new insight into the molecular mechanisms through which DA affects the Toll-like receptor signaling pathway-related immune response and the mechanisms through which lncRNAs regulate mRNA expression. As direct target genes, miR-548s plays important roles in mediating the regulation of FOS by the lncRNA- XLOC_098131 to affect immunity. These data constitute high-quality resources for future genome and functional research.

## Ethics Statement

The experimental animal procedures were approved by the China Agricultural University Animal Care and Use Committee (Beijing, China).

## Author Contributions

HF designed and performed experiments, analyzed data, and wrote the paper. ZeL, LG, BZ, and ZhL performed some experiments, and analyzed some data. CN, JL, and MY analyzed some transcriptome data. BS, GL, and DT analyzed some data. JG, SY, and YW performed some experiments. YG initiated the study, designed animal experiments, analyzed data, and wrote the paper.

### Conflict of Interest Statement

The authors declare that the research was conducted in the absence of any commercial or financial relationships that could be construed as a potential conflict of interest.
