# Supplementary material for: A Novel lncRNA Regulates the Toll-Like Receptor Signaling Pathway and Related Immune Function by Stabilizing FOS mRNA as a Competitive Endogenous RNA
Source: Front Immunol. 2019 Apr 17;10:838. doi: 10.3389/fimmu.2019.00838 (PMC6478817; doi:10.3389/fimmu.2019.00838)
Supplement: Supplementary file 1 [file Table_1.docx]

Table S1. RNA-seq statistics of samples used in the experiment.

| Sample | Original read number | Q30% | Mean quality score (PF) | Clean read numbers | Reads1 number | Reads2 number | | Overall match rate (%) | |
| --- | --- | --- | --- | --- | --- | --- | --- | --- | --- |
| DAD1 | 82746656 | 91.41 | 35.64 | 77448096 | 35948401 | 35845761 | 92.70% | |  |
| DAD2 | 92500088 | 91.42 | 35.64 | 86840638 | 40288053 | 40218776 | 92.70% | |  |
| DAD3 | 93885866 | 91.34 | 35.61 | 87984510 | 40866183 | 40787367 | 92.80% | |  |
| DAD4 | 84365924 | 91.44 | 35.63 | 79053630 | 36517429 | 36434054 | 92.30% | |  |
| DS1 | 79846624 | 91.09 | 35.56 | 74604252 | 34732844 | 34662518 | 93.00% | |  |
| DS2 | 86488342 | 91.34 | 35.6 | 81013370 | 37510698 | 37428011 | 92.50% | |  |
| DS3 | 84191314 | 90.73 | 35.44 | 78295986 | 36165001 | 36072818 | 92.30% | |  |
| DS4 | 84392480 | 90.92 | 35.49 | 78577722 | 36158760 | 36125812 | 92.00% | |  |

DAD, daidzein-deficient group; DS, daidzein-supplemented group. DAD group samples: DAD1-4; DS group samples: DS1-4.
