# Supplementary material for: A Novel lncRNA Regulates the Toll-Like Receptor Signaling Pathway and Related Immune Function by Stabilizing FOS mRNA as a Competitive Endogenous RNA
Source: Front Immunol. 2019 Apr 17;10:838. doi: 10.3389/fimmu.2019.00838 (PMC6478817; doi:10.3389/fimmu.2019.00838)
Supplement: Supplementary file 6 [file Table_6.docx]

Table S6. The full length of XLOC_098131

CTTTCCCTCAAAACCGGTAGGAGAGCCCTGTGCAGGACCTGAGCTGCTCA

CTGAAGGGTGGCTGTGTTCCTATGAGCCACTTTGGTGGGACATGGGTAAG

TCAAGTTGATGGCTGGACTTGATGATCTCGAAGGTCTTTTCCAACCTAAC

CGATCCCATGATTTCCACTCTGCCATCCCACAGAGGTTTTTTCTCTGAGC

TGAGCAGTGGGCCACAGGGCAGTGAATGATGGCAAGGAGATGGAGAGTGA

AGGCACTGTGCCCAAGTCCAGGCGTGAGGAAGCCCCAGTGACTGTGTTTG

CTCACAGCCTGTTTTTCCACTGAATGCTGCCAGCCTCCAGGCTGCTTGAA

CACCACGTGCTGCTTGCATTGCTGTCTTTTCATCTGCCACCCCTTGGTGT

CCCCTGAGGTGTGCGGGCTGGGGATAGCTGCAGGTGACTGGAAATGCTGC

TGGAAAACAACCCCTCATGAGGGAATTAACTCTTGCTTTTATTTCTTTTT

CTGGTGTGAATACAGCTCTGAGGATGCAGGAGAGCTTTGCTGATGTTCCT

TCAGAGCTTGAAATAGTTTTCTTCCCCAAAGATGGATGCTGAGCACTTTC

AAGCTGGCCATTTAGAGAGTTTGTAACCAGAAATCAATCCAGGCAGAAAA

AATGTACATCTACCTGTTTACATCATAAACCACAAACGCGTTTGCATCCT

GCAACAAACACCTGGGCAACATCCAGAGGAGACATTCCCAACGTGTTAAC

TACTGCCAGCGCTGGGATCCTGTAACCACAGCACATGGAGATGATGGGAA

CTCAGTTAAATTAAGGAGAACCACCAAGATTGTGAAACGTGGCAGCAGGA

CAAGAGCTGGTTGCAACACAGGCTAGACTTTAGGTCTGTTGGCCAATCGA

ACACATCTGATGCTGGTGATTGGGATGCCAGAGACAGTTCCCTGACTGCT

GATCTACCCTGTGGGTCACTGAGGCACGTTCTTGAGGGCTGATCCTCCTG

GCCTGCACAAATCTGCATGTCCAATTTCCCGAATGGCAGAAAATTCTACA

TTAAGATGACATTTATCAGCTAATCTTTTAAAAGAAATTAACTGGGTATT

TATGAGGCTTAGACTTAGGGTATCATAGAATCATTAAGGTTGGAAAAGAC

CACCAAGACCAGCAAGTCCGACTGTCAACTGATTCCCCACCATGCCCACC

AACTACATCCTTCTGTGCCACAATCTAACTTCTCAGTGGCACCAGGGAGA

GCACAACCAAGCTCCTTTTGGATCCCACTTGTATCTTGTTCCCTTACTGC

AAGGACAGACCCAAGCCCAACCCTGGCAAACATTTCCTACCAGGGCAGCC

ACCACTGTGCAGGCTGGGTGATGAGGCACCAGTGGGTGCAAGGAAACCCT

AAAGCCCACAGGGGTCAGGGCTGGCAATGCCCCACATCCCTCCATGCTGC

TCAGCCCTTCTGCTGATGTTCTGCAGGAGAGCAGCAGCTGATGGTGCTAA

AAATACCCTTGGGCAGGGTAGCAGTGGTTCAGCACTGGGCTTCCCAGAAG

AGTGCTGGTATTTCAGCCTTGTGACTCCAGCTCAGGCCACTTGATGAACA

GCATGCAACTTTGCTCTGATTTCCCCATTTGGTAGGGAACAATCTCAGTA

ACGGATGGATAAGTCAGCAAACAGCATAGTTCGCTTTCTTATTTTAGGTC

TCCCATAGACATAGATATGAATGACTCTGAAATATAGGCAGCACCTGGTC

CTTCCCTGCTGTCACATTGTCCCCTTGATGGAAAAGCAAAGGAGATTCCT

ATACAAGATCCCTCTGTAGGTCTGAGGAGGCAGCCGTAAATGTCCGACCA

TATGCGTGCCAAACATCCAACCACTCTGCAACCTCACTTTGCCCATTGCA

CGTGGGGCAAGATGGGAGGGATTCCCCAAATCTGCAAGCACATCCCATAA

AATACCCCGGACACCAGCTGAGAGCACCACCTGTCAGTGGAGTGCTTTGT

TCCCAAACCAGGTCCTGCTGGCACATCCCTCTGACACACTCTACAGAAGA

AAAAGTAAAACTAGGAACTTTTTGACTTCTTATTTTGCAGAAGTGTGACC

TTTCGACCTGAATCACGATGAGTGGTTATGAGGAAGAGCTGCTCTCACTT

CTAAATTATTGCCATGCAATGAGCAGGTTTCCATGGGGTAGCTGAAGGCA

TGTCCTGAGGTACAAGGGATACAAGCATCCCATTCTTGCTCTACTTTGCA

CTAAAGGACTGGTCTTAGAGGAGCTTATTCTCTAAAAATGACCTGACTGG

CTGAATAAAACACAATTTCTGTTTTAATATTGTTGTGTTATGAAACACTG

CAATAGAGGCACAAATGTGGGCAAGAACAAGTTTTACTGCAGACGCTTTA

TCTGCTGATAGATTGTCCCAGTGCTGCATTCCTCATTCCCTGGGGGTCTG

GTCCAGCTCAGATTGGAAGATAGTGAAGATCTTGGGAGATCTCCAGTAGT

GTGGGTCAAAATTCACTGATTACAGAAATGCAATTGGTTCAGTTGATCTG

CATAAAGAAAAAAACCACAGCCCATAGTGGGAATGTAGAGTAGGATTGAG

AGGAAAAGCTGAGCCCTAATAGGGAGGCTGGTGCATGGTGTATTCAATAA

TTGCATTTATGAATAAACAATTAAGTATTACCAATGAATCTCTTGCATTT

TATTGACCCAGGCACAGAAGCTGCTTTTTGGCACTGATGACTTGCTCAGT

TCACGCTTGACAATGGGGAATTGTAGTGGCTGTTCCCTTAAGAAATCATC

CAAGATTGGGACTTATTTGTGCGCCATTGCTTGCATCAATTAACAGTACA

ATCTTTTTGTTATGAAAACGCAAACATTAGTAGCCTGAGGAGCCTTGTAA

ATGCAAATATAAAACTTTAATTTCAGTAACCCCCACTTTCCCTTCAAGTA

CAATAAAGGCTCTTTTTTTTTTTTTTTTTCATTTGCTGTTACAACGACGG

GTCTTTATTATTATTATTATTATTTTTCCTCTTTCTCTTCACTTTACATT

GAGTTTAACTCAGGGGATTTCAGTAGGGATAGTTTTCCATCAGCAGATAT

GGGGTGGCTGCAGCCGGTGCTGGGATCTGTGGGCTCCTTGGGGCAGGTCA

GTGCCAACCATAGGGAGGGGATCATGGCTGGGCACTGGGGCCAGCCTTGG

GCAGGGCAGAATTCTCACGTGAGGATTCTTTCCACTCTCTCCAGGCAGCT

GGAGAGCCTAAATTTGGCATCGTGTTTCTGTTGCGAGAATGAGAAGGAGA

GAGAGACCTTGAAACGTGCCC
