# Supplementary material for: A Novel lncRNA Regulates the Toll-Like Receptor Signaling Pathway and Related Immune Function by Stabilizing FOS mRNA as a Competitive Endogenous RNA
Source: Front Immunol. 2019 Apr 17;10:838. doi: 10.3389/fimmu.2019.00838 (PMC6478817; doi:10.3389/fimmu.2019.00838)
Supplement: Supplementary file 7 [file Image_1.pdf]

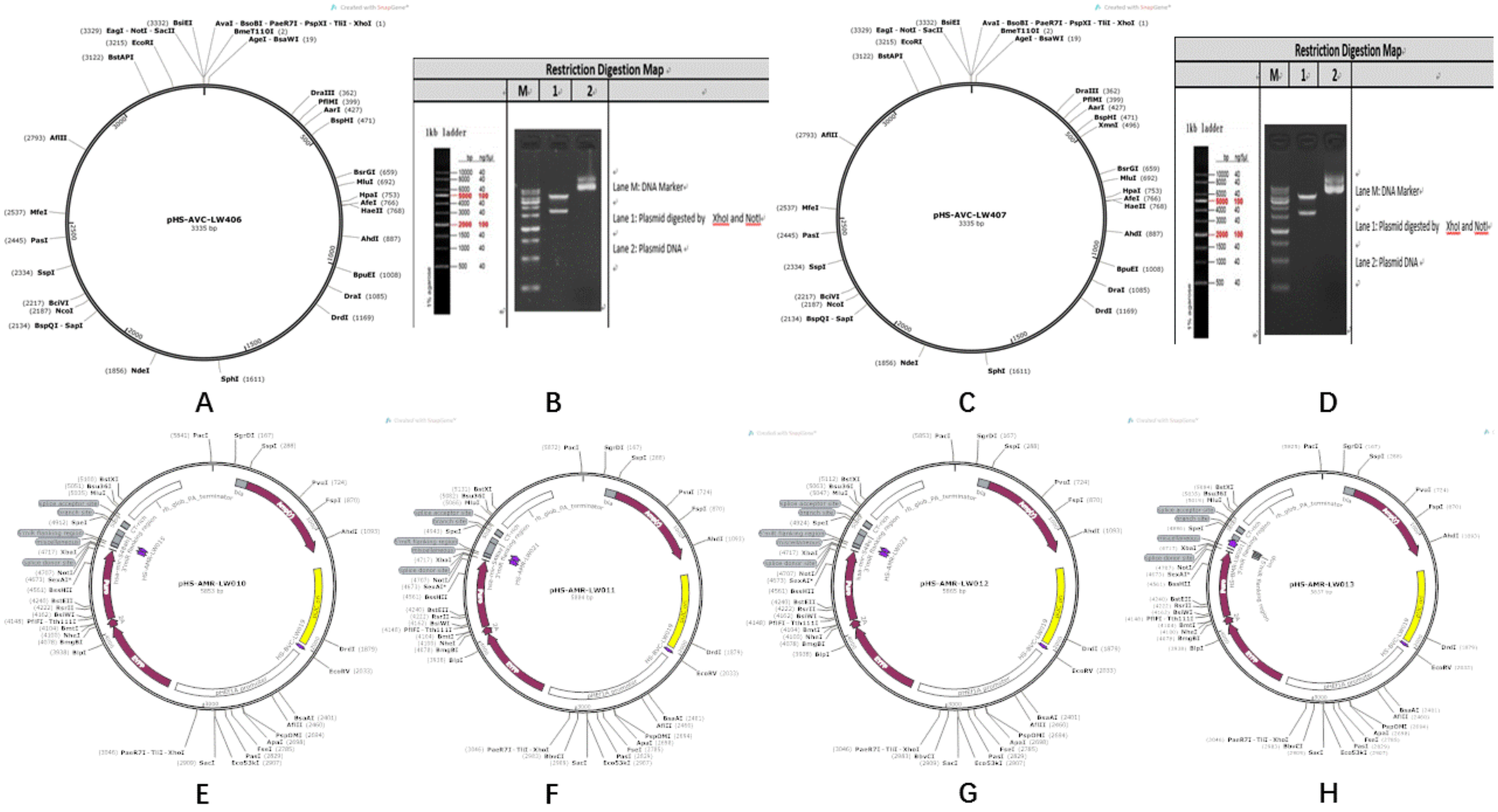

Figure S1. Constructed constructs of the experiment to test the combined ability of XLOC_098131 and mir-548s. (A) lncRNA- XLOC_098131 contains a 3'utr luciferase reporter gene wild-type plasmid named pHS-AVC-LW406 (psi-SV40 promoter-hRluc-XLOC_098131-HSV TK promoter-hluc). (B) Restriction digestion map of pHS-AVC-LW406. (C) A mutant expression plasmid of XLOC_098131 without predicted miR-548s binding sites, named pHS-AVC-

LW407(psi-SV40 promoter-hRluc-XLOC_098131(mutation)-HSV TK promoter-hluc). (D) Restriction digestion map of pHS-AVC-LW407. (E) Overexpression of mir-548s plasmid pHS-AMR-LW010 (pZDonor_hef1a-EYFP-T2A-puro-hsa-mir-548ah). (F) pHS-AMR-LW011 (pZDonor_hef1a-EYFP-T2A-puro-hsa-mir-548ay). (G) pHS-AMR-LW012 (pZDonor_hef1a-EYFP-T2A-puro-hsa-mir-548e). (H) Control mi plasmid pHS-AMR-LW013 (pZDonor_hef1a-EYFP-T2A-puro-control miRNA (FF5))
