# Supplementary material for: A Novel lncRNA Regulates the Toll-Like Receptor Signaling Pathway and Related Immune Function by Stabilizing FOS mRNA as a Competitive Endogenous RNA
Source: Front Immunol. 2019 Apr 17;10:838. doi: 10.3389/fimmu.2019.00838 (PMC6478817; doi:10.3389/fimmu.2019.00838)
Supplement: Supplementary file 9 [file Image_3.pdf]

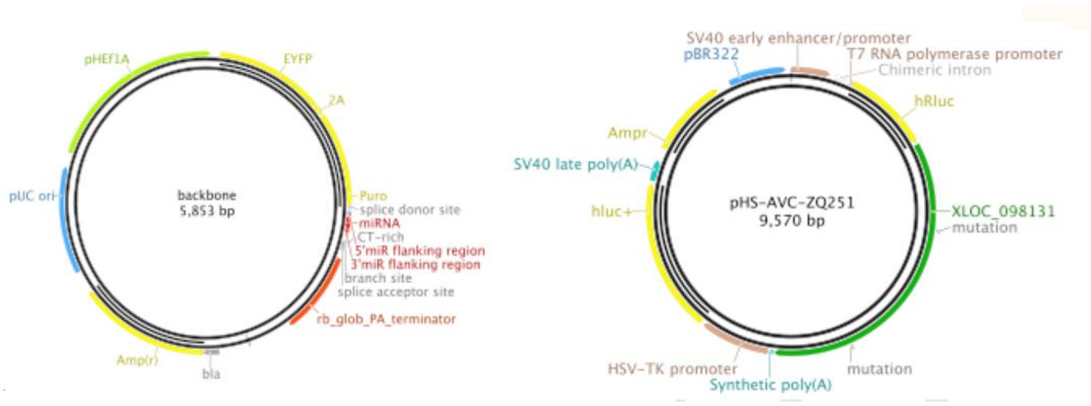

A **B**

Figure S3. Constructed constructs of the experiment to test the combined ability of XLOC_098131 and mir-1180s. (A) Overexpression of mir-1180s plasmid pHS-AMR-ZQ011 (pZDonor_hef1a-EYFP-T2A-puro-hsa-mir-1180-5P) and pHS-AMR-ZQ012 (pZDonor_hef1a-EYFP-T2A-puro-hsa-mir-1180-3P). (B) A mutant expression plasmid of XLOC_098131 without predicted miR-1180s binding sites, named pHS-AVC-LW251 (psi-SV40 promoter-hRluc-XLOC-098131（mutant）-HSV TK promote hluc)
