# Supplementary material for: A Novel lncRNA Regulates the Toll-Like Receptor Signaling Pathway and Related Immune Function by Stabilizing FOS mRNA as a Competitive Endogenous RNA
Source: Front Immunol. 2019 Apr 17;10:838. doi: 10.3389/fimmu.2019.00838 (PMC6478817; doi:10.3389/fimmu.2019.00838)
Supplement: Supplementary file 8 [file Image_2.pdf]

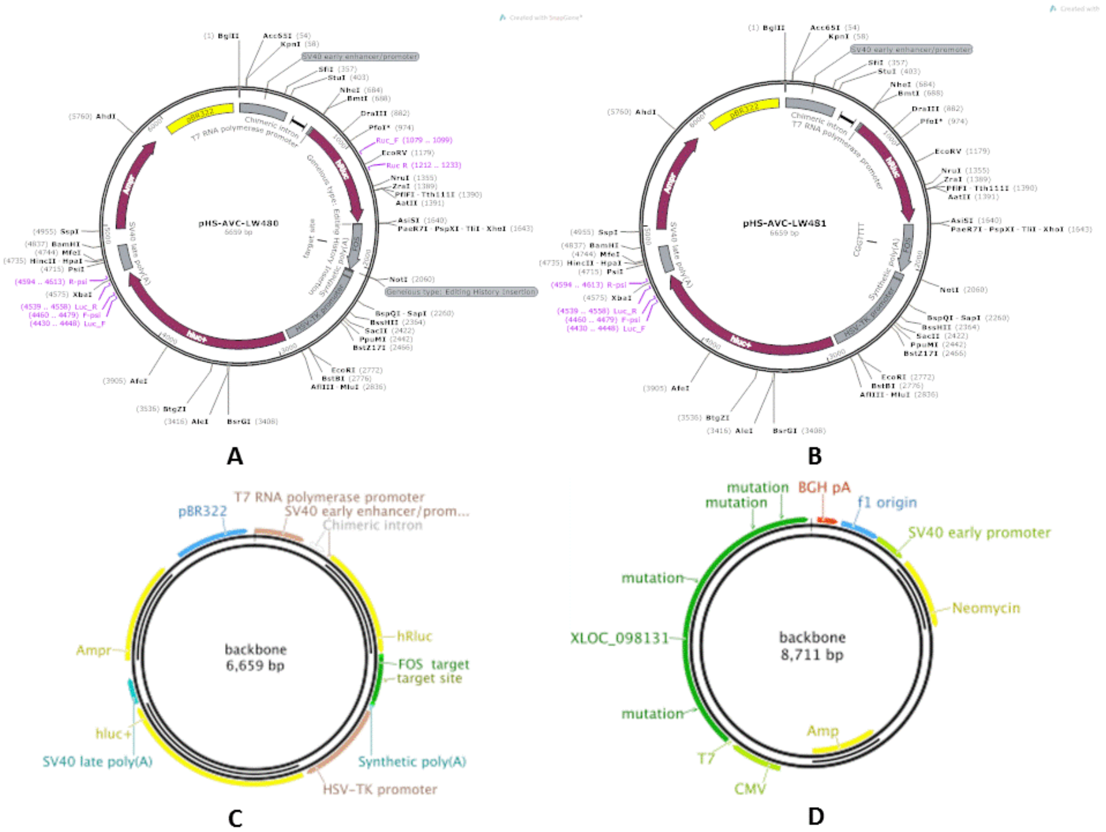

Figure S2. Constructed constructs of the experiment to test the combined ability of FOS and mir-548s, and to verify that XLOC_098131 regulates FOS expression through competitive binding to miR-548s. (A) FOS contains a 3'utr luciferase reporter gene wild-type plasmid named pHS-AVC-LW480 (psi-SV40 promoter-hRluc-Fos gene part (hsa-miRNA-548ay-3p target)-HSV TK promoter-hluc). (B) a mutant expression plasmid of FOS without predicted miR-548s binding sites pHS-AVC-LW481 (psi-SV40 promoter-hRluc-Fos gene part mutation (hsa-miRNA-548ay-3p target mutation)-HSV TK promoter-hluc). (C) FOS luciferase vector Pharma named pHS-AVC-LW482 (psi-SV40 promoter-hRluc-Fos gene part (hsa-miRNA-548ay-3p target)-HSV TK promoter-hluc). (D) XLOC_098131 overexpression plasmid and a mutant overexpression plasmid of XLOC_098131 without predicted miR-548s binding sites.
